# Association of Tumor Necrosis Factor-Alpha, Interleukin-1β, Interleukin-8, and Interferon-γ with Obstructive Sleep Apnea in Both Children and Adults: A Meta-Analysis of 102 Articles

**DOI:** 10.3390/jcm13051484

**Published:** 2024-03-04

**Authors:** Amin Golshah, Edris Sadeghi, Masoud Sadeghi

**Affiliations:** 1 Department of Orthodontics, Kermanshah University of Medical Sciences, Kermanshah 6715847141, Iran; amin.golshah@gmail.com; 2 Medical Biology Research Center, Kermanshah University of Medical Sciences, Kermanshah 6714415185, Iran; sadeghi_mkn@yahoo.com

**Keywords:** cytokine, interleukin, serum, plasma, meta-analysis

## Abstract

**Background**: Cytokines may have a significant impact on sleep regulation. In this meta-analysis, we present the serum/plasma levels of tumor necrosis factor-alpha (TNF-α), interleukin (IL)-8, IL-1β, and interferon-gamma (IFN-γ) in both children and adults with obstructive sleep apnea (OSA) in comparison to controls. **Methods**: Four electronic databases were systematically searched (PubMed, Web of Science, Scopus, and Cochrane Library) through 19 October 2023, without any restrictions on language, date, age, and sex. We used Review Manager version 5.3 to perform meta-analysis and presented the data as standardized mean difference (SMD) and 95% confidence interval (CI) values to evaluate the relationships between the levels of cytokines and OSA. **Results**: A total of 102 articles (150 independent studies) were included in the meta-analysis. The pooled SMDs in adults were 1.42 (95%CI: 1.11, 1.73; *p* < 0.00001), 0.85 (95%CI: 0.40, 1.31; *p* = 0.0002), 0.69 (95%CI: 0.22, 1.16; *p* = 0.004), and 0.39 (95%CI: −0.37, 1.16; *p* = 0.31) for TNF-α, IL-8, IL-1β, and IFN-γ, respectively. The pooled SMDs in children were 0.84 (95%CI: 0.35, 1.33; *p* = 0.0008), 0.60 (95%CI: 0.46, 0.74; *p* < 0.00001), 0.25 (95%CI: −0.44, 0.93; *p* = 0.49), and 3.70 (95%CI: 0.75, 6.65; *p* = 0.01) for TNF-α, IL-8, IL-1β, and IFN-γ, respectively. **Conclusions**: The levels of proinflammatory cytokines of TNF-α, IL-8, and IL-1β in adults, and TNF-α, IL-8, and IFN-γ in children with OSA, are significantly higher than those in controls.

## 1. Introduction

Obstructive sleep apnea (OSA) is a common disorder characterized by episodes of partial or complete upper airway obstruction during sleep, often resulting in disrupted sleep and other health issues [1,2]. In the general population, nearly 1 billion adults aged 30–69 years worldwide have mild-to-severe OSA, and 425 million people worldwide have moderate-to-severe OSA [3]. Polysomnography is the standard diagnostic test for OSA in both adults and children [4]. The apnea–hypopnea index (AHI), where mild OSA has an AHI from 5 to <15, moderate has an AHI from 15 to 30, and severe has an AHI of >30, with this value representing the average number of apneas and hypopneas that occur per hour of sleep, is quantified during this test [5].

The occurrence of OSA in adults varies. It is estimated that about 15 to 30 percent of males and 10 to 15 percent of females have OSA when it is broadly defined as an AHI of 5 or more events/h [6,7,8]. However, when stricter definitions are applied (such as AHI ≥ 5 events/h with symptoms or AHI ≥ 15 events/h), the estimated prevalence drops to around 15% and 5% in males and females, respectively [6,7,9]. In children, the prevalence of OSA is estimated to range from 0.7% to 13% [10]. This broad range can be attributed to the different thresholds used to define OSA in children and the various diagnostic methods used [11]. Typically, OSA in children is defined as an AHI of 1 or more events/h [12,13].

In patients suffering from OSA, biopsies of the upper airway tissue have shown signs of subepithelial edema and an overabundance of inflammatory cell infiltration [14,15]. Elevated levels of inflammatory biomarkers have been detected in these patients [16,17,18,19,20], which have been observed to decrease with effective treatment [16,17,21]. Cytokines, a varied group of non-antibody proteins that signal between cells, play a crucial role in managing local and systemic immune and inflammatory responses, as well as numerous other biological processes. These proteins may have a significant impact on sleep regulation [22,23].

Meta-analyses have reported on the blood levels of tumor necrosis factor-alpha (TNF-α) in adults. These meta-analyses were conducted on OSA [24,25,26], interleukin (IL-8 in adults [27], IL-8 and IL-1β in both children and adults [28], TNF-α and IL-8 in adults [29], IL-8 in both children and adults [30], and TNF-α in both children and adults [20] in comparison to control groups. The outcomes of these meta-analyses varied, as did the results reported in the original articles [31,32,33,34,35]. The conflicting results in these meta-analyses could be due to several factors such as differences in study populations (age, ethnicity, and health status), variations in the methodologies used in the studies (measurement techniques and statistical analyses), and the inherent biological variability in cytokine levels. It is also important to note that cytokine levels can be influenced by many factors, including time of day, diet, stress, and other environmental factors. Therefore, it is not surprising to see some degree of variability in the results of these studies. In this meta-analysis, we investigated the difference between serum/plasma levels of TNF-α, IL-8, IL-1β, and interferon-gamma (IFN-γ) in both children and adults with OSA compared to controls. We used more studies than previous meta-analyses and introduced some new analyses. In addition, we performed subgroup analyses on variables such as ethnicities, mean BMI, age, mean AHI, sample size, and blood sample. The selection of these specific cytokines is based on their known roles in inflammation and immune response, which are key processes involved in the pathophysiology of OSA. Normal ranges of TNF-α, IL-8, IL-1β, and IFN-γ are 0–2.2 pg/mL, 0–66.1 pg/mL, ≤6.7 pg/mL, and 0–8.41 pg/mL, respectively (https://healthmatters.io/, accessed on 30 January 2024).

## 2. Materials and Methods

### 2.1. Literature Search

Four electronic databases were systematically searched (PubMed, Web of Science, Scopus, and Cochrane Library) through 19 October 2023, without any restrictions. The search terms were (“OSA” or “OSAS” or “sleep apnea” or “obstructive sleep apnea” or “obstructive sleep apnea syndrome” or “OSAHS” or “obstructive sleep apnea/hypopnea syndrome”) and (“interleukin-1β” or “interleukin-1beta” or “IL-1β” or “IL-1beta” or “IL1beta” OR “IL1β” or “interleukin-8” or “IL-8” or “tumor necrosis factor” or “TNFα” or “TNF-alpha” or “TNFalpha” or “TNFα” or “interferon gamma” or “interferon-γ” or “IFN-γ” or “IFN-gamma” or “IFNgamma” or “IFNγ”) and (“serum” or “plasma” or “blood” or “circulating”). We also went through the references of eligible studies and manually reviewed articles to identify possible relevant publications, as well as going through electronic sources such as Google Scholar.

### 2.2. Study Selection

The PICOS framework was introduced as the inclusion criteria (Population (P): both children and adults diagnosed with OSA. Intervention (I): measurement of TNF-α, IL-8, IL-1β, and IFN-γ levels. Comparison (C): comparison of these levels with a control group of both children and adults without OSA. Outcome (O): the association of TNF-α, IL-8, IL-1β, and IFN-γ levels with OSA. Study design (S): observational studies). OSA patients were diagnosed according to the clinical practice guidelines from the American College of Physicians used for adults [36] and children [37]. The levels of cytokines were determined using pg/mL or calculated in relation to the accordant unit.

The inclusion criteria involved: (1) Studies that included adult participants with an AHI ≥ 5 events/h as adults with OSA and AHI < 5 events/h as adults without OSA and child participants with an AHI ≥ 1 events/h as children with OSA and AHI < 1 events/h as children without OSA. (2) Studies that used morning levels of cytokines. (3) OSA was diagnosed via polysomnography.

The exclusion criteria included the following: (1) Participants with a history or diagnosis of any systemic diseases overlapping with OSA (e.g., other respiratory, cardiovascular, and endocrine diseases); patients who received nutritional support or underwent therapy (e.g., medication, operation, continuous positive airway pressure); case reports or those articles lacking statistical data; and articles without a control group. (2) Articles selected an adult control group with an AHI > 5 events/h or a child control group with an AHI > 1 event/h. (3) Reviews, meta-analyses, letters to the editors, and book chapters. (4) Articles without any data; articles which measured the level of cytokines in saliva, urine, exhaled breath condensate, and cells; and articles which measured the overnight or evening levels of cytokines. (5) Gene expression and a high sensitivity level of cytokines.

### 2.3. Data Extraction

Two authors (M.S. and E.S.) independently screened the literature and extracted data to ensure that the screening core criteria and data gathering were consistent. If the opinions were different, further discussion was carried out or an additional person (A.G.) was invited to participate in discussions until a consensus was reached. We extracted the first author, year of publication, nationality, sample size, gender, mean BMI, mean age, mean AHI, blood sample, and levels of cytokines. The quality of the included studies was evaluated according to the Newcastle–Ottawa scale (NOS) [38]. Two authors independently performed the process of screening title/abstract and screening full texts, and a consensus was reached on all decisions.

### 2.4. Statistical Analysis and Data Synthesis

We used Review Manager version 5.3 for meta-analysis and presented the data as standardized mean difference (SMD) and 95% confidence interval (CI) in order to evaluate the relationship between the levels of cytokines and OSA. We utilized GetData Graph Digitizer software version 2.26 to report mean ± SD on the graphs. In some studies, median (interquartile) or median (range) was reported, which we converted into mean ± standard deviation (SD). We converted standard error (SE) into SD by SE = $\frac{SD}{\sqrt{N}}$, N = Number of cases. We extracted mean ± SD from log (mean ± SD) in “https://brilliant.org/wiki/log-normal-distribution/”, accessed on 30 January 2024.

The heterogeneity of the studies was assessed using the I2 statistic, with the level of significance set at *p* < 0.05. Given the probable heterogeneity of the studies, a random-effects model was employed in the meta-analysis when *P*_heterogeneity_ was less than 0.10 (I^2^ more than 50%) [39]. Otherwise, a fixed-effect model was used [40]. Publication bias was evaluated using a funnel plot and Begg’s and Egger’s tests, with the level of significance set at *p* < 0.10. Comprehensive meta-analysis version 2.0 (CMA 2.0) software was used to perform bias analyses, meta-regression, and sensitivity analyses.

We conducted a trial sequential analysis (TSA) using TSA software (version 0.9.5.10 beta) [41]. The required information size (RIS) was calculated for blood cytokine levels, with alpha risk set at 5% and beta risk at 20%. The mean difference was based on empirical assumptions. If the Z-curve interrupted the RIS, sufficient cases were entered into the studies and the conclusion could be reliable.

## 3. Results

### 3.1. Search Strategy

**Figure 1:**
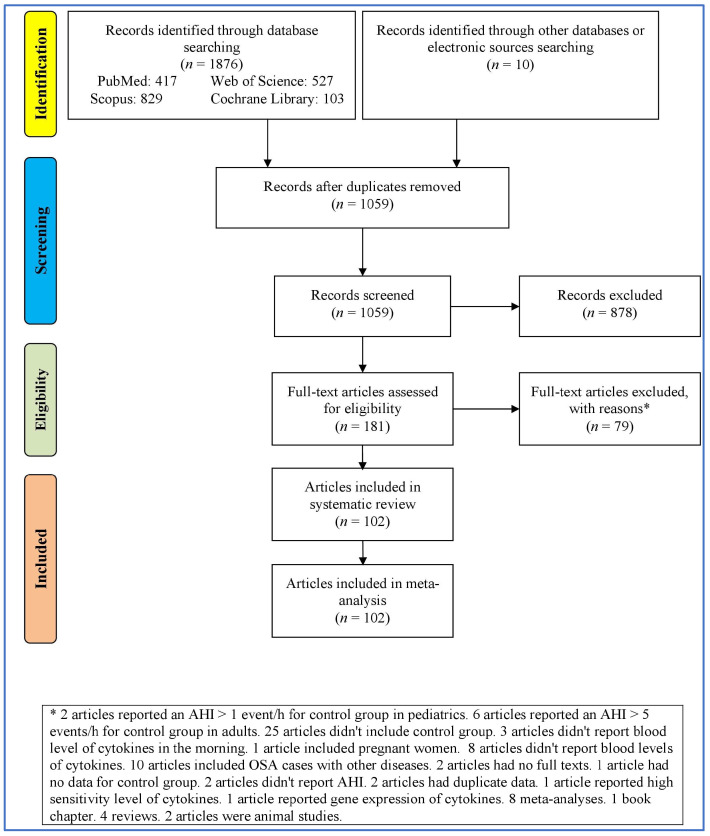
Flowchart of the study selection.

Records were identified through database searching (1876 records) and the use of other sources (10 records). After removing duplicates, there were 1059 unique records, all of which were screened. Full-text articles were assessed for eligibility (181 articles). However, due to various reasons, only 102 articles were included in the systematic review. All of these 102 articles [8,16,21,31,32,33,42,43,44,45,46,47,48,49,50,51,52,53,54,55,56,57,58,59,60,61,62,63,64,65,66,67,68,69,70,71,72,73,74,75,76,77,78,79,80,81,82,83,84,85,86,87,88,89,90,91,92,93,94,95,96,97,98,99,100,101,102,103,104,105,106,107,108,109,110,111,112,113,114,115,116,117,118,119,120] also appeared to be included in a meta-analysis (Figure 1).

### 3.2. Characteristics of Articles

Out of all articles included in the meta-analysis, eighty-five articles were reported that dealt with adults [8,16,21,31,32,33,42,43,44,45,46,47,48,49,50,51,52,53,54,55,56,57,58,59,60,61,62,63,64,65,66,67,68,69,70,71,72,73,74,75,76,77,78,79,80,81,82,83,84,85,86,87,88,89,90,91,92,93,94,95,96,97,98,99,100,101,102,103,104,105,106,107,108,109,110,111,112,113,114,115,116,117,118,119,120] while seventeen considered children [12,13,34,35,121,122,123,124,125,126,127,128,129,130,131,132,133]. The studies are from various countries, including Egypt, China, Iran, USA, Saudi Arabia, Greece, Spain, India, Turkey, Brazil, Italy, Poland, Hungary, and France. The ethnicity of the participants varies across the studies, including Arab, Caucasian, Asian, and mixed ethnicities. The number of cases and controls in each study varies. The variables measured in the studies include apnea–hypopnea index (AHI), age, and body mass index (BMI) for both cases and controls. The samples used for the studies are mostly serum and plasma (Table 1 and Table 2). Each study has been assigned a quality score (Supplementary File S1).

### 3.3. Pooled Analysis in Adults

**Figure 2:**
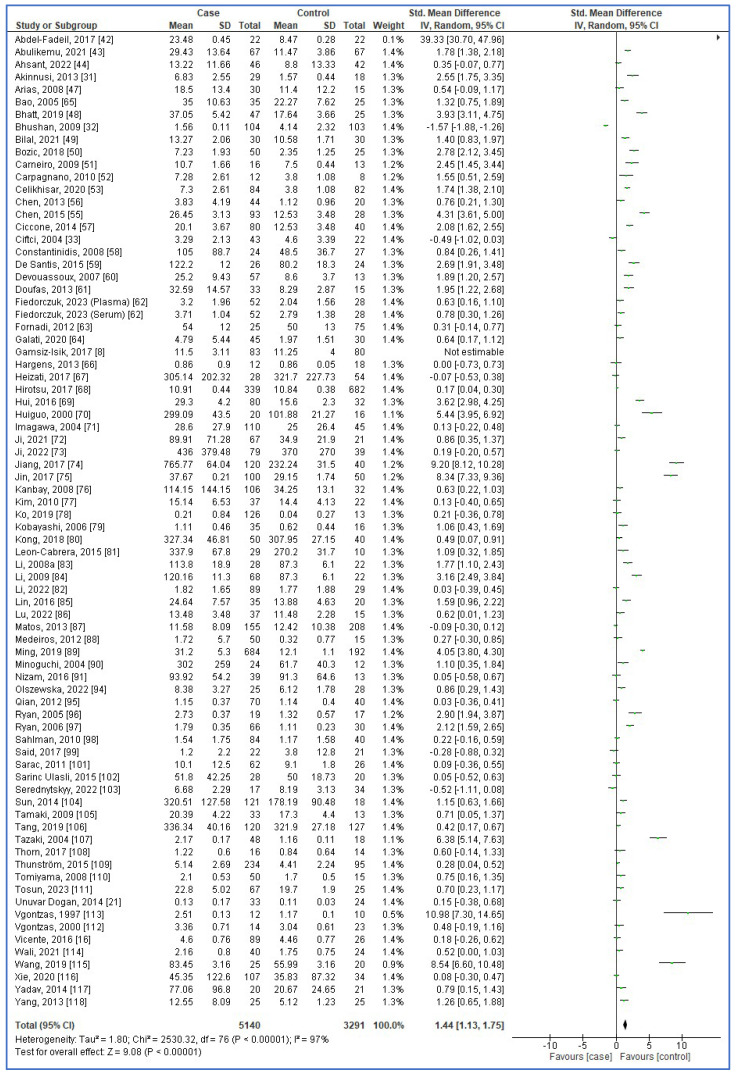
Forest plot analysis of comparison of serum/plasma levels of TNF-α in adults with OSA compared to controls. Each square represents the result of an individual study. The size of the square indicates the weight of the study in the meta-analysis. Horizontal lines represent the confidence intervals of the individual studies. The longer the lines, the wider the confidence interval, indicating less reliability of the study results. The diamond represents the pooled result from all the studies. The width of the diamond indicates the 95% confidence interval for the pooled result.

**Figure 3:**
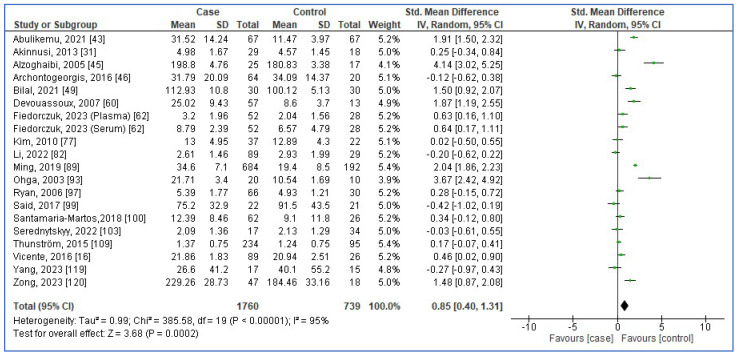
Forest plot analysis of comparison of serum/plasma levels of IL-8 in adults with OSA compared to controls. Each square represents the result of an individual study. The size of the square indicates the weight of the study in the meta-analysis. Horizontal lines represent the confidence intervals of the individual studies. The longer the lines, the wider the confidence interval, indicating less reliability of the study results. The diamond represents the pooled result from all the studies. The width of the diamond indicates the 95% confidence interval for the pooled result.

**Figure 4:**
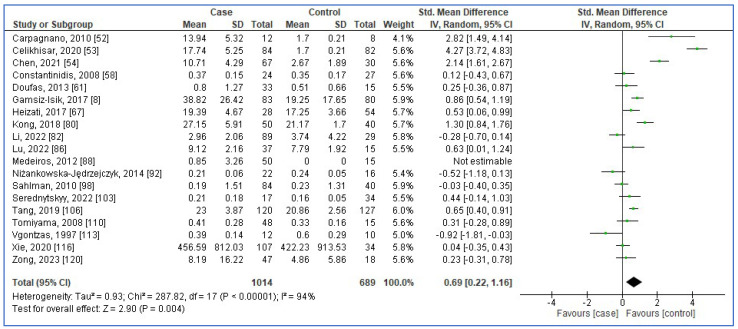
Forest plot analysis of comparison of serum/plasma levels of IL-1β in adults with OSA compared to controls. Each square represents the result of an individual study. The size of the square indicates the weight of the study in the meta-analysis. Horizontal lines represent the confidence intervals of the individual studies. The longer the lines, the wider the confidence interval, indicating less reliability of the study results. The diamond represents the pooled result from all the studies. The width of the diamond indicates the 95% confidence interval for the pooled result.

**Figure 5:**
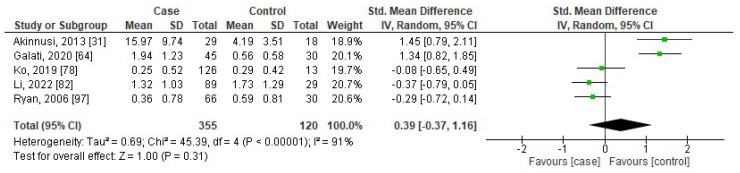
Forest plot analysis of comparison of serum/plasma levels of IFN-γ in adults with OSA compared to controls. Each square represents the result of an individual study. The size of the square indicates the weight of the study in the meta-analysis. Horizontal lines represent the confidence intervals of the individual studies. The longer the lines, the wider the confidence interval, indicating less reliability of the study results. The diamond represents the pooled result from all the studies. The width of the diamond indicates the 95% confidence interval for the pooled result.

Figure 2, Figure 3, Figure 4 and Figure 5 show forest plots of the association between the blood levels of TNF-α, IL-8, IL-1β, and IFN-γ in adults with OSA compared to controls. The pooled SMDs were 1.42 (95%CI: 1.11, 1.73; *p* < 0.00001; I^2^ = 97%), 0.85 (95%CI: 0.40, 1.31; *p* = 0.0002; I^2^ = 95%), 0.69 (95%CI: 0.22, 1.16; *p* = 0.004; I^2^ = 94%), and 0.39 (95%CI: −0.37, 1.16; *p* = 0.31; I^2^ = 91%) for TNF-α, IL-8, IL-1β, and IFN-γ, respectively. The results reported that levels of TNF-α, IL-8, and IL-1β in adults with OSA were higher than seen in controls.

### 3.4. Pooled Analysis in Children

**Figure 6:**
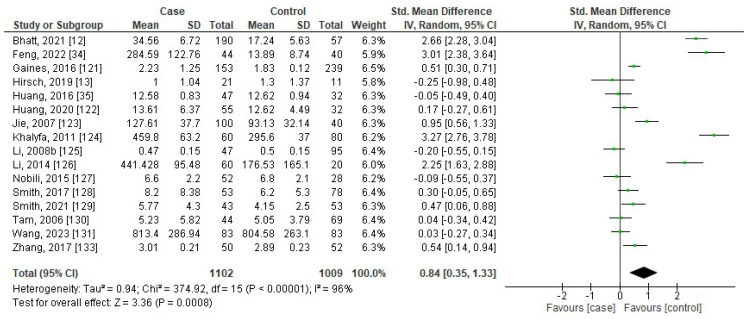
Forest plot analysis of comparison of serum/plasma levels of TNF-α in children with OSA compared to controls. Each square represents the result of an individual study. The size of the square indicates the weight of the study in the meta-analysis. Horizontal lines represent the confidence intervals of the individual studies. The longer the lines, the wider the confidence interval, indicating less reliability of the study results. The diamond represents the pooled result from all the studies. The width of the diamond indicates the 95% confidence interval for the pooled result.

**Figure 7:**
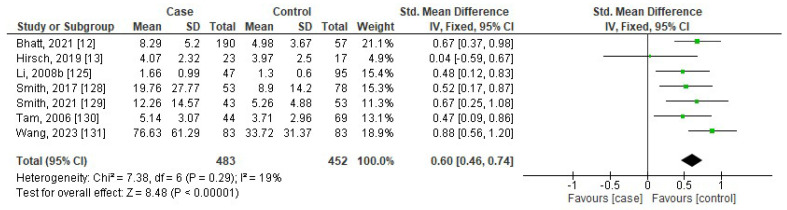
Forest plot analysis of comparison of serum/plasma levels of IL-8 in children with OSA compared to controls. Each square represents the result of an individual study. The size of the square indicates the weight of the study in the meta-analysis. Horizontal lines represent the confidence intervals of the individual studies. The longer the lines, the wider the confidence interval, indicating less reliability of the study results. The diamond represents the pooled result from all the studies. The width of the diamond indicates the 95% confidence interval for the pooled result.

**Figure 8:**
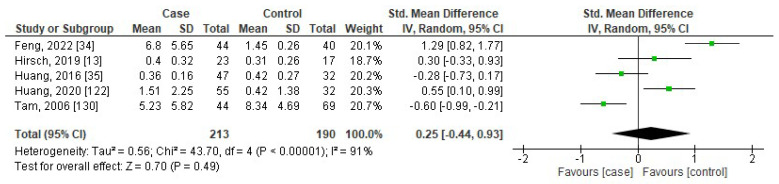
Forest plot analysis of comparison of serum/plasma levels of IL-1β in children with OSA compared to controls. Each square represents the result of an individual study. The size of the square indicates the weight of the study in the meta-analysis. Horizontal lines represent the confidence intervals of the individual studies. The longer the lines, the wider the confidence interval, indicating less reliability of the study results. The diamond represents the pooled result from all the studies. The width of the diamond indicates the 95% confidence interval for the pooled result.

**Figure 9:**
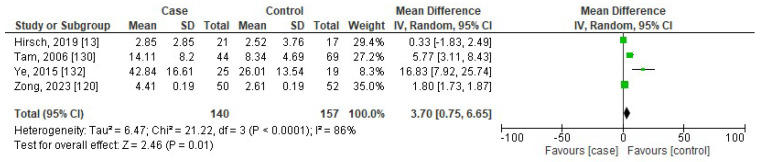
Forest plot analysis of comparison of serum/plasma levels of IFN-γ in children with OSA compared to controls. Each square represents the result of an individual study. The size of the square indicates the weight of the study in the meta-analysis. Horizontal lines represent the confidence intervals of the individual studies. The longer the lines, the wider the confidence interval, indicating less reliability of the study results. The diamond represents the pooled result from all the studies. The width of the diamond indicates the 95% confidence interval for the pooled result.

Figure 6, Figure 7, Figure 8 and Figure 9 show the forest plots of association between blood levels of TNF-α, IL-8, IL-1β, and IFN-γ in children with OSA compared to controls. The pooled SMDs were 0.84 (95%CI: 0.35, 1.33; *p* = 0.0008; I^2^ = 96%), 0.60 (95%CI: 0.46, 0.74; *p* < 0.00001; I^2^ = 19%), 0.25 (95%CI: −0.44, 0.93; *p* = 0.49; I^2^ = 91%), and 3.70 (95%CI: 0.75, 6.65; *p* = 0.01; I^2^ = 86%) for TNF-α, IL-8, IL-1β, and IFN-γ, respectively. The results reported that levels of TNF-α, IL-8, and IFN-γ in children with OSA were higher than in controls.

### 3.5. Subgroup Analysis

Table 3 shows the subgroup analysis of several variables affecting TNF-α, IL-8, and IL-1β levels in adults and TNF-α levels in children with OSA compared to controls. The results recommended that ethnicity was an effective factor for all factors, and also that blood samples and mean BMI played roles for IL-8 and IL-1β levels in adults, for sample size, mean age, and mean AHI for IL-8 and IL-1β in adults, and for TNF-α levels in children.

### 3.6. Subgroup Analysis

Table 4 shows a random meta-regression analysis for cytokines, with seven variables used.

For IL-8 in adults, the coefficient is 0.0737 for mean AHI in these cases, indicating that for each unit increase in AHI, the expected change in IL-8 is an increase of 0.0737 units. In these cases, there is a 0.2613 mean BMI in controls, indicating that for each unit increase in BMI in controls, the expected change in IL-8 is a decrease of 0.2613 units. There is a 0.1160 mean age in controls, indicating that for each unit increase in age in controls, the expected change in IL-8 is an increase of 0.1160 units. The *p*-value is less than 0.05, indicating that these effects are statistically significant.

For TNF-α in children, the coefficient is 0.0524 for mean AHI in cases, indicating that for each unit increase in AHI in cases, the expected change in TNF-α is an increase of 0.0524 units when holding all other variables constant. The *p*-value is 0.0314, which is less than 0.05, indicating that this effect is statistically significant.

For IL-8 in children, all variables in this category are statistically significant, with *p*-values less than 0.05. This means that all these variables, namely publication year, sample size, mean BMI in cases, mean age in cases, mean AHI in cases, mean BMI in controls, and mean age in controls, are confounding factors for the IL-8 biomarker in children’s cases.

### 3.7. Publication Bias

Table 5 presents the results of the publication bias analysis for various biomarkers in both adult and child populations. The analysis was conducted using two tests: Egger’s test and Begg’s test. A *p*-value of less than 0.10 is considered statistically significant and indicates potential publication bias. In this case, the biomarker of TNF-α (adult) shows significant publication bias in both tests, with *p*-values less than 0.0001. The biomarker of IL-8 (children) also shows potential publication bias in Egger’s test, with a *p*-value of 0.0507, but not in Begg’s test. All other biomarkers do not show statistically significant publication bias in either of the tests. Supplementary File S2 shows the funnel plots.

### 3.8. Sensitivity Analysis

The pooled results were stable for four cytokines in both adults and children, because “one-study-removed” and “cumulative” analyses did not change the results.

### 3.9. TSA

The Z-curve did not cross the RIS for IFN-γ in adults and IL-1β in children, indicating that the evidence is not yet sufficient to confirm an effect and that more trials are needed. However, for the other biomarkers, the Z-curve crossed the RIS, suggesting that enough evidence was accumulated to make conclusions. Please refer to Supplementary File S3 for the specific TSA plots.

## 4. Discussion

The meta-analysis presents a comparison between blood levels of TNF-α, IL-8, IL-1β, and IFN-γ in OSA in adults and children, comparing patients with OSA and healthy controls. The results show higher levels of TNF-α, IL-8, and IL-1β in adults with OSA, and higher levels of TNF-α, IL-8, and IFN-γ in children with OSA compared to controls. Subgroup analysis revealed that ethnicity, blood sample, mean BMI, sample size, mean age, and mean AHI are significant factors influencing these levels. Meta-regression analysis further confirmed these findings, with all variables showing statistical significance for IL-8 in children. Publication bias analysis indicated a potential bias for TNF-α in adults and IL-8 in children. The TSA suggested that more trials are needed for IFN-γ in adults and IL-1β in children, while sufficient evidence has been gathered for the other biomarkers.

The development of OSA is a complex process involving multiple factors, one of which includes the selective activation of inflammatory response pathways [134]. Studies have shown that intermittent hypoxia, a condition often seen in OSA, can trigger inflammatory pathways, leading to cardiovascular or metabolic diseases [135]. Factors such as oxidative stress, cardiovascular inflammation, endothelial dysfunction, and metabolic abnormalities in OSA could hasten the process of atherogenesis [136]. The regulation of systemic and airway inflammation in OSA could vary depending on the type of cytokine, and could be influenced by OSA itself and concurrent obesity [137].

In cases of OSA, there is an observed increase in the levels of proinflammatory cytokines and a decrease in anti-inflammatory factors, ultimately leading to endothelial dysfunction [138]. The current meta-analysis identifies all the mentioned cytokines, namely TNF-α, IL-8, IL-1β, and IFN-γ, as proinflammatory cytokines [139]. The results indicate elevated levels of TNF-α, IL-8, and IL-1β in adults with OSA, and increased levels of TNF-α, IL-8, and IFN-γ in children with OSA when compared to control groups. These cytokines are pivotal in regulating immune responses and inflammation.

A review highlighted that patients with OSA are more prone to coronary atherosclerosis and to exhibit signs of cardiac remodeling and dysfunction [140]. In 2020, another study affirmed that OSA could be viewed as a systemic inflammatory disease, with an imbalance of non-neuronal cholinergic and pro/anti-inflammatory cytokines playing a role in the onset and progression of comorbidities in OSA patients [141]. A 2020 review discovered that the recurring episodes of airway collapse and obstruction in OSA patients lead to apnea and arousal during sleep, which results in intermittent hypoxia and excessive daytime sleepiness, thereby contributing to inflammation [142]. These studies underscore the connection between OSA and inflammation, suggesting that the effective management of OSA could help to mitigate inflammation and enhance patient outcomes. However, the precise mechanisms are intricate and continue to be the subject of ongoing research.

A meta-analysis by Imani in 2020 [20] found that factors such as ethnicity, BMI, AHI, age, and sample size significantly influenced the blood levels of TNF-α in OSA patients compared to control groups. Two other meta-analyses highlighted that the severity of OSA also impacts the blood levels of TNF-α [24,26]. Another meta-analysis by Li et al. [30] indicated that age and ethnicity play a role in the relationship between OSA and IL-8 levels. The current meta-analysis reaffirms that factors like ethnicity, age, BMI, AHI, and sample size have varying effects on different cytokines. These variations could be attributed to the unique mechanisms of each cytokine, which can be further investigated in the future. The present meta-analysis reported that as the AHI score increased, the IL-8 levels in adults and children and TNF-α level in children significantly increased. As the age increased, the IL-8 levels in adults and children significantly increased. In addition, as the sample size and BMI increased, the IL-8 levels in children significantly increased. The results suggest that the levels of cytokines, which are small proteins important in cell signaling, vary among different ethnicities. It is important to note that many factors can contribute to these differences, including genetic factors, environmental influences, lifestyle choices, and more. It is also crucial to remember that while such research can identify trends across populations, individual health outcomes can vary widely within any ethnic group. Therefore, these findings should be interpreted with caution and used as a starting point for further research, rather than as definitive conclusions about individual health.

### 4.1. Strengths

(1) This study includes a systematic review of 102 articles, providing a broad overview of the research in this field. (2) This study conducts subgroup analysis for TNF-α, IL-8, and IL-1β levels in adults and TNF-α levels in children with OSA compared to controls. This allows for a more nuanced understanding of the effects of these variables. (3) This study performs meta-regression analysis for cytokines with seven variables, providing insights into the relationships between these variables and the cytokines. (4) This study conducts a publication bias analysis, ensuring the robustness of the results. (5) Sensitivity analyses reports the stability of pooled results.

### 4.2. Limitations

(1) Heterogeneity was high for most of the analyses. This suggests that there was considerable variability in the results of the included studies. (2) The publication bias analysis showed significant bias for the biomarker TNF-α in adults and potential bias for the biomarker IL-8 in children. This could affect the reliability of the results. (3) The Z-curve did not cross the RIS for IFN-γ in adults and IL-1β in children, indicating that the evidence is not yet sufficient to confirm an effect and that more trials are needed. (4) Whatever we selected as the morning levels of cytokines, the measurements were not conducted at identical times or with the same sample, biasing the possible association. (5) Although we tried to exclude some comorbidities that can influence the results, it is impossible to be sure that they were not influenced by others which we did not refer to.

## 5. Conclusions

This systematic review and meta-analysis of 102 articles provides substantial evidence that levels of proinflammatory cytokines TNF-α, IL-8, and IL-1β in adults and TNF-α, IL-8, and IFN-γ in children with OSA are higher than in controls. This study also identifies ethnicity, blood sample, mean BMI, sample size, mean age, and mean AHI as significant factors influencing these cytokine levels.

These findings underscore the role of inflammation in OSA and highlight the potential of these cytokines as biomarkers for disease severity and progression. This could guide the development of targeted therapies and improve patient management strategies.

While this study provides valuable insights, it also reveals areas for future research. The evidence for IFN-γ in adults and IL-1β in children is not yet sufficient, indicating a need for more trials. Furthermore, the presence of publication bias for TNF-α (adult) and IL-8 (children) underscores the importance of publishing all results, regardless of their significance, to ensuring a comprehensive understanding of the disease. Future studies should also explore the mechanisms underlying the identified associations and their implications for patient outcomes. This will help in the development of more effective personalized treatment strategies for OSA.

## Figures and Tables

**Table 1:** Characteristics of the articles including Adults.

First Author, Publication Year	Country	Ethnicity	Case/Control No.	Variable	Case			Control			Sample	Quality Score
					AHI, Events/h	Age, Years	BMI, kg/m^2^	AHI, Events/h	Age, Years	BMI, kg/m^2^		
Abdel-Fadeil, 2017 [42]	Egypt	Arab	22/22	TNF-α	32.17 ± 4.39	49.92 ± 2.10	36.00 ± 1.10	3.72 ± 0.36	47.55 ± 2.35	36.62 ± 1.14	Serum	7
Abulikemu, 2021 [43]	China	Caucasian	67/67	TNF-α, IL-8	27.34 ± 4.87	47.51 ± 9.64	24.13 ± 2.97	4.31 ± 1.05	45.93 ± 10.01	23.94 ± 2.85	Serum	8
Ahsant, 2022 [44]	Iran	Asian	46/42	TNF-α	36.75 ± 22.19	59.38 ± 9.37	36.05 ± 6.75	0.00 ± 0.00	52.79 ± 5.76	33.00 ± 4.64	Serum	8
Akinnusi, 2013 [31]	USA	Mixed	29/18	TNF-α, IL-8, IFN-γ	32.2 ± 13.1	54.5 ± 8.9	31.1 ± 5.7	1.9 ± 1.6	52.33 ± 9.3	29.5 ± 5.1	Serum	8
Alzoghaibi, 2005 [45]	Saudi Arabia	Arab	25/17	IL-8	73.5 ± 6.9	49.5 ± 2.2	36.3 ± 1.5	<5	30.7 ± 1.5	23.4 ± 0.7	Serum	7
Archontogeorgis, 2016 [46]	Greece	Caucasian	64/20	IL-8	≥5	51.78 ± 11.55	36.34 ± 13.18	<5	51.40 ± 16.24	33.73 ± 5.67	Serum	8
Arias, 2008 [47]	Spain	Caucasian	30/15	TNF-α	43.8 ± 27.0	52.0 ± 13.0	30.5 ± 4.0	3.7 ± 3.3	48.0 ± 10.0	28.7 ± 4.7	Plasma	8
Bao, 2005 [65]	China	Asian	35/25	TNF-α	≥5	50 ± 10	29.24 ± 3.24	3.67 ± 0.53	42.56 ± 16.93	22.90 ± 2.58	Serum	7
Bhatt, 2019 [48]	India	Asian	47/25	TNF-α	13.5 ± 6.4	44.2 ± 9.1	32.5 ± 6.9	2.3 ± 1.1	28.5 ± 8.6	41 ± 8.5	Serum	7
Bhushan, 2009 [32]	India	Asian	104/103	TNF-α	47.90 ± 24.60	46.18 ± 10.70	31.48 ± 4.26	2.80 ± 1.70	40.00 ± 10.00	30.94 ± 4.27	Plasma	8
Bilal, 2021 [49]	Turkey	Caucasian	30/30	TNF-α, IL-8	24.65 ± 5.74	26.2 ± 1.34	30.41 ± 6.15	2.62 ± 1.34	42.53 ± 9.81	29.09 ± 4.52	Serum	8
Bozic, 2018 [50]	Croatia	Caucasian	50/25	TNF-α	35.0 ± 11.0	53.0 ± 11.9	28.9 ± 2.7	<5	52.5 ± 10.2	27.8 ± 2.2	Plasma	8
Carneiro, 2009 [51]	Brazil	Mixed	16/13	TNF-α	65.7 ± 9.9	40.1 ± 2.8	46.9 ± 2.0	3.2 ± 0.5	38.8 ± 3.3	42.8 ± 1.3	Plasma	7
Carpagnano, 2010 [52]	Italy	Caucasian	12/8	TNF-α, IL-1β	48.8 ± 23.1	47.3 ± 13.2	42.6 ± 6.8	3.2 ± 0.9	42 ± 4	24.6 ± 2.6	Plasma	7
Celikhisar, 2020 [53]	Turkey	Caucasian	84/82	TNF-α, IL-1β	27.4 ± 18.6	50.9 ± 5.7	32.4 ± 6	1.8 ± 1.4	49.3 ± 5.8	30.6 ± 5.6	Serum	8
Chen, 2013 [56]	China	Asian	44/20	TNF-α	14.56 ± 2.85	27.12 ± 3.53	24.56 ± 2.85	3.30 ± 0.90	42.00 ± 11.00	26.00 ± 3.30	Plasma	7
Chen, 2015 [55]	China	Asian	93/28	TNF-α	27.00 ± 4.06	42.28 ± 8.55	28.84 ± 3.82	2.60 ± 1.20	43.70 ± 9.80	26.40 ± 2.50	Plasma	8
Chen, 2021 [54]	China	Asian	67/30	IL-1β	37.53 ± 9.51	43.72 ± 10.75	31.33 ± 6.05	3.06 ± 1.35	44.77 ± 10.67	26.15 ± 3.55	Serum	8
Ciccone, 2014 [57]	Italy	Caucasian	80/40	TNF-α	33.9 ± 21.0	52.8 ± 10.6	28.6 ± 3.0	2.1 ± 1.1	52.3 ± 10.5	28.2 ± 2.7	Plasma	8
Ciftci, 2004 [33]	Turkey	Caucasian	43/22	TNF-α	33.20 ± 5.00	49.60 ± 9.10	31.90 ± 4.10	1.50 ± 0.96	47.20 ± 10.30	31.00 ± 3.10	Serum	8
Constantinidis, 2008 [58]	Greece	Caucasian	24/27	TNF-α, IL-1β	23.3 ± 3.6	45.1 ± 8.2	≥25	3.5 ± 0.4	45.1 ± 8.2	≥25	Serum	8
De Santis, 2015 [59]	Italy	Caucasian	26/24	TNF-α	26.1 ± 12.1	41.8 ± 7.4	33.0 ± 5.2	1.6 ± 0.9	43.7 ± 8.2	30.8 ± 4.3	Serum	8
Devouassoux, 2007 [60]	France	Caucasian	57/13	IL-8	41 ± 14.3	54 ± 11	28.7 ± 5.4	<5	45 ± 7	28.2 ± 3.7	Plasma	7
Doufas, 2013 [61]	USA	Mixed	33/15	TNF-α, IL-1β	18.07 ± 14.66	34.56 ± 8.41	26.11 ± 3.13	2.41 ± 1.41	32.55 ± 9.47	24.69 ± 3.45	Serum	8
Fiedorczuk, 2023 [62]	Poland	Caucasian	52/28	TNF-α, IL-8	28.87 ± 3.55	43.68 ± 12.10	28.87 ± 3.56	2.36 ± 1.69	40.12 ± 12.68	26.69 ± 2.92	Serum & Plasma	8
Fornadi, 2012 [63]	Hungary	Caucasian	25/75	TNF-α	≥5	54 ± 12	29 ± 5	<5	50 ± 13	26 ± 5	Serum	8
Galati, 2020 [64]	Italy	Caucasian	45/30	TNF-α, IFN-γ	≥5	53.9 ± 11.6	28 ± 2.2	<5	55.0 ± 5.8	26.3 ± 1.8	Serum	9
Gamsiz-Isik, 2017 [8]	Turkey	Caucasian	83/80	TNF-α, IL-1β	≥5	46.87 ± 8.21	31.53 ± 3.44	<5	37.53 ± 24.38	44.23 ± 9.83	Serum	7
Hargens, 2013 [66]	USA	Mixed	12/18	TNF-α	25.4 ± 5.4	22.8 ± 0.8	32.4 ± 1.0	2.1 ± 0.3	21.9 ± 0.6	29.3 ± 0.5	Serum	8
Heizati, 2017 [67]	China	Asian	28/54	TNF-α, IL-1β	38.03 ± 42.72	44.00 ± 8.26	26.09 ± 1.75	6.75 ± 10.82	44.94 ± 8.33	25.30 ± 1.79	Serum	9
Hirotsu, 2017 [68]	Brazil	Mixed	339/682	TNF-α	19.3 ± 9.44	50.8 ± 13.2	29.6 ± 5.8	2.5 ± 10.4	38.2 ± 12.7	25.4 ± 3.8	Serum	7
Hui, 2016 [69]	China	Asian	80/32	TNF-α	55.3 ± 18.7	46.3 ± 5.9	NA	<5	41.2 ± 3.7	NA	Serum	7
Huiguo, 2000 [70]	China	Asian	20/16	TNF-α	44.0 ± 21.0	47.4 ± 13.6	27.6 ± 3.3	4.29 ± 2.16	47.6 ± 14.7	23.1 ± 3	Plasma	8
Imagawa, 2004 [71]	Japan	Asian	110/45	TNF-α	≥5	NA	27.7 ± 4.4	<5	NA	22.9 ± 2.9	Serum	7
Ji, 2021 [72]	China	Asian	67/21	TNF-α	38.42 ± 11.60	44.15 ± 14.69	32.87 ± 8.47	2.69 ± 0.35	42.97 ± 10.26	31.57 ± 8.85	Serum	8
Ji, 2022 [73]	China	Asian	79/21	TNF-α	29.46 ± 6.67	58.55 ± 10.46	25.30 ± 3.26	2.53 ± 1.38	56.10 ± 12.40	24.50 ± 3.10	Serum	8
Jiang, 2017 [74]	China	Asian	120/40	TNF-α	46.60 ± 4.56	24.82 ± 10.70	28.50 ± 5.13	2.13 ± 1.26	46.50 ± 12.30	27.50 ± 6.20	Plasma	7
Jin, 2017 [75]	China	Asian	100/50	TNF-α	38.01 ± 8.04	55.28 ± 7.13	26.75 ± 3.50	3.62 ± 1.54	56.13 ± 6.21	25.19 ± 2.45	Plasma	8
Kanbay, 2008 [76]	Turkey	Caucasian	106/32	TNF-α	40.14 ± 14.30	51.39 ± 10.37	31.06 ± 5.87	1.96 ± 1.08	44.79 ± 13.35	28.25 ± 5.49	Serum	8
Kim, 2010 [77]	Korea	Asian	37/22	TNF-α, IL-8	43.39 ± 18.08	41.03 ± 11.75	17.66 ± 3.68	1.25 ± 1.25	26.00 ± 6.91	23.88 ± 2.30	Serum	7
Ko, 2019 [78]	China	Asian	126/13	TNF-α, IFN-γ	36.09 ± 16.58	45.80 ± 13.02	27.42 ± 3.93	1.83 ± 1.34	35.92 ± 7.69	24.1 ± 2.33	Serum	7
Kobayashi, 2006 [79]	Japan	Asian	35/16	TNF-α	52.26 ± 14.76	51.40 ± 13.10	27.90 ± 3.60	<5	41.00 ± 13.10	27.40 ± 3.70	Serum	8
Kong, 2018 [80]	China	Asian	50/40	TNF-α, IL-1β	37.34 ± 19.02	54.34 ± 14.38	26.86 ± 3.12	3.31 ± 1.09	50.42 ± 8.35	22.2 ± 3.5	Serum	8
Leon-Cabrera, 2015 [81]	Mexico	Mixed	29/10	TNF-α	51.4 ± 25.7	37.2 ± 11.4	45.2 ± 8.4	7.5 ± 3.3	43.4 ± 11.5	23.6 ± 2.1	Serum	7
Li, 2008a [83]	China	Asian	28/22	TNF-α	33.4 ± 28.6	45.1 ± 10.2	27.7 ± 4.5	2.9 ± 1.3	43 ± 9	23.3 ± 2.0	Serum	8
Li, 2009 [84]	China	Asian	68/22	TNF-α	38.91 ± 3.08	45.29 ± 10.91	27.75 ± 4.56	2.09 ± 1.30	43 ± 93	23.3 ± 2.00	Serum	8
Li, 2022 [82]	China	Asian	89/29	TNF-α, IL-8 IL-1β	18.86 ± 17.23	45.87 ± 5.17	29.05 ± 4.89	2.28 ± 0.85	45.38 ± 5.39	30.30 ± 5.30	Plasma	9
Lin, 2016 [85]	Taiwan	Asian	35/20	TNF-α	59.3 ± 23.2	46.0 ± 7.0	29.2 ± 1.9	3.6 ± 0.8	59.3 ± 23.2	43.0 ± 8.0	Serum	8
Lu, 2022 [86]	China	Asian	37/15	TNF-α, IL-1β	27.26 ± 9.46	51.73 ± 11.09	24.67 ± 3.09	1.95 ± 1.35	51.73 ± 11.09	24.67 ± 3.09	Serum	9
Matos, 2013 [87]	Brazil	Mixed	155/208	TNF-α	≥5	51.2	29.7	<5	40.7	24.5	Plasma	8
Medeiros, 2012 [88]	Brazil	Mixed	50/15	TNF-α, IL-1β	>5	64.29 ± 7.73	28.62 ± 4.01	<5	62.50 ± 8.40	25.81 ± 4.04	Serum	8
Ming, 2019 [89]	China	Asian	684/192	TNF-α, IL-8	31.15 ± 9.12	51.34 ± 5.16	≤30	4.34 ± 2.01	52.18 ± 4.51	≤30	Serum	7
Minoguchi, 2004 [90]	Japan	Asian	24/12	TNF-α	34.1 ± 14.7	50.1 ± 11.7	29.1 ± 2.2	2.3 ± 1.9	48.1 ± 10.5	25.3 ± 1.2	Serum	7
Nizam, 2016 [91]	Turkey	Caucasian	39/13	TNF-α	45.6 ± 20.7	47.3 ± 10.4	33.2 ± 56.4	2.6 ± 1.8	43.2 ± 9.1	31.7 ± 4.5	Serum	8
Niżankowska-Jędrzejczyk, 2014 [92]	Poland	Caucasian	22/16	IL-1β	23.62 ± 12.32	52.50 ± 8.33	30.15 ± 2.77	1.90 ± 2.78	54.50 ± 8.33	28.02 ± 3.36	Plasma	9
Ohga, 2003 [93]	Japan	Asian	20/10	IL-8	38.5 ± 3.1	47.8 ± 2.2	29.4 ± 1.4	3.1 ± 0.4	48.9 ± 2.9	28.4 ± 2.9	Serum	9
Olszewska, 2022 [94]	Poland	Caucasian	25/18	TNF-α	34.9 ± 17.3	50.2 ± 11.3	34.1 ± 3.7	1.5 ± 0.8	33.0 ± 14.0	NA	Serum	7
Qian, 2012 [95]	China	Asian	70/40	TNF-α	≥5	45.8 ± 8.2	28.9 ± 2.3	<5	46.3 ± 8.1	24.1 ± 2.3	Serum	8
Ryan, 2005 [96]	Ireland	Caucasian	19/17	TNF-α	49.6 ± 25.2	39.5 ± 2.0	32.3 ± 16.1	1.03 ± 0.5	39.5 ± 19.5	31.1 ± 15.5	Serum	8
Ryan, 2006 [97]	Ireland	Caucasian	66/30	TNF-α, IL-8, IFN-γ	35.0 ± 13.9	42.5 ± 8.5	32.5 ± 4.8	1.2 ± 1.0	41.0 ± 8.0	30.7 ± 3.1	Serum	8
Sahlman, 2010 [98]	Finland	Caucasian	84/40	TNF-α, IL-1β	9.6 ± 2.9	50.4 ± 9.3	32.5 ± 3.3	1.9 ± 1.4	45.6 ± 11.5	31.5 ± 3.5	Plasma	8
Said, 2017 [99]	Oman	Caucasian	22/21	TNF-α, IL-8	≥ 30	40.4 ± 8.6	NA	<5	33.9 ± 6.7	NA	Plasma	7
Santamaria-Martos, 2018 [100]	Spain	Caucasian	228/132	IL-8	19.48 ± 44.52	61.40 ± 14.16	28.39 ± 4.12	1.89 ± 1.74	44.35 ± 11.24	24.59 ± 3.22	Serum	7
Sarac, 2011 [101]	Turkey	Caucasian	62/26	TNF-α	29.5 ± 1.9	50.0 ± 19.7	33.7 ± 4.2	<5	49.7 ± 11.1	34.3 ± 5.4	Plasma	7
Sarinc Ulasli, 2015 [102]	Turkey	Caucasian	28/20	TNF-α	30.58 ± 18.39	51.70 ± 10.20	32.40 ± 5.60	2.24 ± 0.99	45.30 ± 14.00	30.40 ± 8.00	Serum	8
Serednytskyy, 2022 [103]	Spain	Caucasian	17/34	TNF-α, IL-8, L-1β	8.94 ± 5.90	37.60 ± 4.04	28.80 ± 6.06	0.54 ± 0.54	35.36 ± 5.42	26.04 ± 4.57	Serum	8
Sun, 2014 [104]	China	Asian	121/18	TNF-α	40.8 ± 10.9	43.3 ± 11.6	27.1 ± 3.1	2.1 ± 1.8	43.9 ± 13.4	25.7 ± 3.8	Serum	8
Tamaki, 2009 [105]	Japan	Asian	33/13	TNF-α	39.35 ± 12.05	53.30 ± 49.60	39.35 ± 4.25	3.80 ± 1.80	35.50 ± 9.70	23.60 ± 2.60	Serum	7
Tang, 2019 [106]	China	Asian	120/127	TNF-α, IL-1β	39.00 ± 18.38	48.88 ± 9.76	26.86 ± 3.12	3.31 ± 1.09	47.37 ± 9.12	22.50 ± 3.30	Serum	8
Tazaki, 2004 [107]	Japan	Asian	48/18	TNF- α	36.05 ± 1.75	50.60 ± 4.80	28.90 ± 1.60	3.70 ± 0.40	48.20 ± 3.00	27.80 ± 0.80	Serum	8
Thorn, 2017 [108]	UK	Caucasian	16/14	TNF-α	30.0 ± 18.0	59.0 ± 13.0	32.7 ± 4.0	0.0 ± 0.0	58.0 ± 7.0	30.6 ± 2.7	Serum	8
Thunström, 2015 [109]	Sweden	Caucasian	234/95	TNF-α, IL-8	28.9 ± 13.7	65.3 ± 7.1	26.8 ± 2.1	3.1 ± 1.3	61.4 ± 9.5	25.2 ± 2.5	Serum	9
Tomiyama, 2008 [110]	Japan	Asian	50/15	TNF-α, IL-1β	42.7 ± 27.9	51.4 ± 13.0	26.9 ± 4.2	<5	53.0 ± 10.0	24.3 ± 2.5	Plasma	8
Tosun, 2023 [111]	Turkey	Caucasian	67/25	TNF-α	39.06 ± 15.55	49.24 ± 10.12	32.47 ± 4.80	2.70 ± 1.40	40.40 ± 13.00	28.40 ± 4.60	Serum	7
Unuvar Dogan, 2014 [21]	Turkey	Caucasian	33/24	TNF-α	47.2 ± 23.2	45.3 ± 8.5	31.0 ± 1.7	3.6 ± 1.8	40.5 ± 9.5	30.7 ± 1.5	Serum	8
Vgontzas, 1997 [113]	USA	Mixed	12/10	TNF-α, IL-1β	63.7 ± 10.3	40.9 ± 2.2	40.5 ± 3.2	0.0 ± 0.0	24.1 ± 0.8	24.6 ± 0.7	Plasma	6
Vgontzas, 2000 [112]	USA	Mixed	14/23	TNF-α	48.7 ± 5.6	46.6 ± 3.0	38.4 ± 1.6	0.88 ± 0.4	43.6 ± 2.5	30.7 ± 1.6	Plasma	8
Vicente, 2016 [16]	Spain	Caucasian	89/26	TNF-α, IL-8	28.00 ± 23.70	45.33 ± 14.81	30.03 ± 5.04	1.90 ± 2.70	45.00 ± 11.11	28.70 ± 4.37	Plasma	8
Wali, 2021 [114]	Saudi Arabia	Arab	40/24	TNF-α	36.74 ± 23.60	47.50 ± 13.18	37.50 ± 11.40	2.90 ± 2.00	31.70 ± 11.70	30.00 ± 8.60	Serum	7
Wang, 2019 [115]	China	Asian	25/20	TNF-α	27.90 ± 5.95	62.10 ± 4.40	30.15 ± 2.50	1.80 ± 0.54	63.60 ± 5.70	24.40 ± 3.23	Serum	8
Xie, 2020 [116]	China	Asian	107/34	TNF-α, IL-1β	40.49 ± 12.69	48.22 ± 17.25	27.85 ± 3.07	2.23 ± 1.49	34.74 ± 14.02	23.80 ± 4.00	Serum	7
Yadav, 2014 [117]	UK	Caucasian	20/21	TNF-α	27.26 ± 25.60	49.00 ± 10.00	52.00 ± 6.00	4.98 ± 2.47	45.00 ± 9.00	50.00 ± 8.00	Serum	8
Yang, 2013 [118]	China	Asian	25/25	TNF-α	24.00 ± 17.00	54.00 ± 7.00	27.39 ± 2.91	3.00 ± 1.00	53.00 ± 7.00	26.22 ± 1.90	Plasma	8
Yang, 2023 [119]	Canada	Mixed	17/15	IL-8	43.0 ± 29.0	62.0 ± 9.3	30.0 ± 4.5	7.2 ± 4.5	60.0 ± 7.8	25.0 ± 2.7	Serum	8
Zong, 2023 [120]	China	Asian	47/18	IL-8, IL-1β	33.09 ± 12.07	50.32 ± 12.45	27.41 ± 3.43	1.90 ± 1.20	52.90 ± 16.90	25.70 ± 3.08	Serum	9

**Table 2:** Characteristics of articles including children.

First Author, Publication Year	Country	Ethnicity	Case/Control No.	Variable	Case			Control			Sample	Quality Score
					AHI, Events/h	Age, Years	BMI, kg/m^2^	AHI, Events/h	Age, Years	BMI, kg/m^2^		
Bhatt, 2021 [12]	India	Asian	190/57	TNF-α, IL-8	≥1	10.70 ± 3.00	27.1 ± 6.53	<1	11.80 ± 2.60	27.4 ± 4.88	Serum	9
Feng, 2022 [34]	China	Asian	44/40	TNF-α, IL-1β	11.59 ± 9.01	6.66 ± 1.96	16.70 ± 2.85	0.63 ± 0.46	6.90 ± 1.83	16.22 ± 2.39	Serum	9
Gaines, 2016 [121]	USA	Mixed	153/239	TNF-α	13.78 ± 4.73	17.70 ± 2.20	NA	0.89 ± 4.77	16.40 ± 2.10	NA	Plasma	7
Hirsch, 2019 [13]	Australia	Mixed	21/11, 23/17, 23/17, 21/20	TNF-α, IL-8, IL-1β, IFN-γ	≥1	10.0 ± 1.7	NA	<1	10.7 ± 1.2	NA	Serum	7
Huang, 2016 [35]	Taiwan	Asian	47/32	TNF-α, IL-1β	9.13 ± 1.67	7.84 ± 0.56	16.95 ± 0.47	0.41 ± 0.07	7.02 ± 0.65	6.55 ± 0.58	Plasma	7
Huang, 2020 [122]	Taiwan	Asian	55/32	TNF-α, IL-1β	15.71 ± 22.60	7.67 ± 2.64	16.83 ± 4.03	0.46 ± 0.28	7.02 ± 0.65	17.44 ± 3.08	Plasma	9
Jie, 2007 [123]	China	Asian	100/40	TNF-α	≥1	4.67	NA	<1	NA	NA	Serum	6
Khalyfa, 2011 [124]	USA	Mixed	60/80	TNF-α	8.9 ± 2.7	7.2 ± 0.2	NA	0.5 ± 0.2	7.2 ± 0.3	NA	Plasma	8
Li, 2008b [125]	China	Asian	47/95	TNF-α, IL-8	14.1 ± 8.0	11.1 ± 1.3	NA	0.7 ± 0.6	10.7 ± 1.3	NA	Serum	7
Li, 2014 [126]	China	Asian	60/20	TNF-α	≥1	5.51 ± 2.01	11.98 ± 2.18	<1	5.66 ± 2.39	15.78 ± 1.97	Plasma	8
Nobili, 2015 [127]	Italy	Caucasian	52/28	TNF-α	4.99 ± 3.07	11.30 ± 2.10	28.30 ± 4.90	0.58 ± 0.30	11.70 ± 1.90	26.4 ± 5.9	Serum	9
Smith, 2017 [128]	USA	Mixed	53/78	TNF-α, IL-8	11.29 ± 8.00	9.21 ± 2.63	22.43 ± 10.39	0.40 ± 0.30	9.70 ± 2.50	19.4 ± 4.4	Plasma	7
Smith, 2021 [129]	USA	Mixed	43/53	TNF-α, IL-8	10.3 ± 9.1	9.0 ± 2.6	20.4 ± 5.3	0.8 ± 1.4	10.0 ± 2.3	20.1 ± 4.8	Serum	8
Tam, 2006 [130]	Australia	Mixed	44/69	TNF-α, IL-8, IL-1β, IFN-γ	5.3 ± 6.5	7.3 ± 3.7	19.4 ± 5.5	0.0 ± 0.0	7.6 ± 4.0	17.9 ± 3.9	Serum	9
Wang, 2023 [131]	China	Asian	83/83	TNF-α, IL-8	7.9 ± 8.4	7.0 ± 2.7	17.1 ± 3.3	0.0 ± 0.0	6.8 ± 3.5	16.9 ± 3.4	Serum	9
Ye, 2015 [132]	China	Asian	25/19	IFN-γ	34.76 ± 15.28	6.45 ± 2.84	NA	0.38 ± 0.20	6.63 ± 2.71	NA	Serum	7
Zhang, 2017 [133]	China	Asian	50/52	TNF-α, IFN-γ	≥1	6.6	NA	<1	6.4	NA	Serum	7

NA: not available. IL: interleukin. TNF-α: tumor necrosis factor-alpha. IFN-γ: interferon-gamma. AHI: apnea–hypopnea index. BMI: body mass index.

**Table 3:** Subgroup analysis.

Biomarker	Subgroup	Variable, N	SMD	95%CI		* p * -Value	I^2^
TNF-α (adult)	Ethnicity	Asian (35)	1.95	1.32	2.58	**<0.00001**	98%
		Caucasian (31)	0.82	0.53	1.10	**<0.00001**	91%
		Arab (2)	19.68	−18.36	57.71	0.31	99%
		Mixed (10)	1.08	0.55	1.61	**<0.0001**	93%
	Blood sample	Serum (55)	1.20	0.88	1.53	**<0.00001**	96%
		Plasma (23)	2.00	1.25	2.74	**<0.00001**	98%
	Sample size	≥100 (24)	1.41	0.81	2.01	**<0.00001**	99%
		<100 (54)	1.37	1.06	1.68	**<0.00001**	92%
	Mean BMI, kg/m^2^	≥30 (20)	0.86	0.30	1.41	**0.002**	96%
		<30 (35)	1.33	0.95	1.71	**<0.00001**	96%
	Mean age, years	≥50 (13)	1.88	0.83	2.93	**0.00005**	99%
		<50 (44)	1.43	1.00	1.86	**<0.00001**	96%
	Mean AHI in cases, event/h	≥30 (44)	1.88	1.33	2.42	**<0.00001**	98%
		<30 (23)	1.23	0.8	1.64	**<0.00001**	5%
IL-8 (adult)	Ethnicity	Asian (5)	1.33	0.14	2.5	**0.03**	97%
		Caucasian (12)	0.60	0.20	0.99	**0.003**	89%
		Mixed (2)	0.03	−0.42	0.48	0.88	21%
	Blood sample	Serum (15)	0.99	0.45	1.54	**0.0004**	96%
		Plasma (5)	0.45	−0.21	1.11	0.18	88%
	Sample size	≥100 (5)	0.88	−0.12	1.88	0.08	98%
		<100 (15)	0.82	0.36	1.28	**0.0004**	89%
	Mean BMI, kg/m^2^	≥30 (2)	0.11	−0.22	0.44	0.51	29%
		<30 (10)	0.97	0.45	1.49	**0.0003**	91%
	Mean age, years	≥50 (6)	0.60	−0.35	1.56	0.22	98%
		<50 (12)	0.93	0.38	1.49	**0.001**	92%
	Mean AHI in cases, event/h	≥30 (10)	1.23	0.44	2.02	**0.002**	96%
		<30 (9)	0.60	0.15	1.04	**0.008**	90%
IL-1β (adult)	Ethnicity	Asian (9)	0.61	0.17	1.04	**0.006**	88%
		Caucasian (6)	1.22	−0.09	2.53	0.07	97%
		Mixed (3)	−0.28	−1.43	0.86	0.63	78%
	Blood sample	Serum (12)	1.00	0.39	1.61	**0.001**	95%
		Plasma (7)	0.11	−0.39	0.60	0.67	79%
	Sample size	≥100 (6)	0.90	−0.05	1.86	0.06	98%
		<100 (13)	0.57	0.10	1.04	**0.02**	87%
	Mean BMI, kg/m^2^	≥30 (2)	1.88	−2.82	6.58	0.43	99%
		<30 (9)	0.50	0.22	0.78	**0.0004**	66%
	Mean age, years	≥50 (5)	0.41	−0.19	1.01	0.18	82%
		<50 (10)	0.55	0.10	1.01	**0.02**	89%
	Mean AHI in cases, event/h	≥30 (9)	0.73	0.23	1.23	**0.005**	89%
		<30 (6)	0.79	−0.59	2.17	0.26	97%
TNF-α (children)	Ethnicity	Asian (9)	1.03	0.27	1.78	**0.008**	97%
		Caucasian (1)	−0.09	−0.55	0.37	0.70	-
		Mixed (6)	0.72	−0.04	1.48	0.06	96%
	Blood sample	Serum (10)	0.71	0.06	1.36	**0.03**	96%
		Plasma (6)	1.06	0.21	1.91	**0.01**	97%
	Sample size	≥100 (9)	0.89	0.23	1.54	**0.008**	97%
		<100 (7)	0.78	−0.04	1.60	0.06	94%
	Mean age, years	≥9 (7)	0.50	−0.19	1.19	0.16	96%
		<9 (8)	1.14	0.27	2.01	**0.010**	97%
	Mean AHI in cases, event/h	≥10 (7)	0.56	0.04	1.08	**0.03**	93%
		<10 (5)	0.63	−0.44	1.70	0.25	97%

Bold number means statistically significant (*p* < 0.05). IL: interleukin. TNF-α: tumor necrosis factor-alpha. AHI: apnea–hypopnea index. BMI: body mass index. In table, mean BMI and mean age included both groups (cases and controls) together. N: number of studies.

**Table 4:** Random meta-regression analysis.

Biomarker	Variable	Coefficient	Standard Error	95% Lower	95% Upper	Z-Value	2-Sided *p*-Value
TNF-α (adult)	Publication year	0.0007	0.0006	−0.0005	0.0019	1.11	0.2685
	Sample size	−0.0028	0.0035	−0.0097	0.0041	−0.80	0.4237
	Mean BMI in cases, kg/m^2^	−0.0058	0.0381	−0.0805	0.0690	−0.15	0.8796
	Mean age in cases, year	−0.0237	0.0217	−0.0663	0.0189	−1.09	0.2752
	Mean AHI in cases, events/h	0.0280	0.0170	−0.0053	0.0614	1.65	0.0995
	Mean BMI in controls, kg/m^2^	−0.0081	0.0342	−0.0750	0.0589	−0.24	0.8131
	Mean age in controls, year	0.0242	0.0129	−0.0010	0.0495	1.88	0.0598
IL-8 (adult)	Publication year	0.0009	0.0017	−0.0023	0.0041	0.55	0.5810
	Sample size	−0.0073	0.0039	−0.0151	0.0004	−1.86	0.0623
	Mean BMI in cases, kg/m^2^	0.0666	0.0685	−0.0676	0.2008	0.97	0.3306
	Mean age in cases, year	−0.0535	0.0331	−0.1183	0.0114	−1.62	0.1059
	Mean AHI in cases, events/h	0.0737	0.0238	0.0270	0.1204	3.09	**0.0020**
	Mean BMI in controls, kg/m^2^	−0.2613	0.1304	−0.5169	−0.0056	−2.00	**0.0452**
	Mean age in controls, year	0.1160	0.0441	0.0296	0.2024	2.63	**0.0085**
IL-1β (adult)	Publication year	−0.0018	0.0026	−0.0069	0.0033	−0.69	0.4891
	Sample size	0.0083	0.0071	−0.0057	0.0223	1.17	0.2437
	Mean BMI in cases, kg/m^2^	0.1050	0.1128	−0.1161	0.3262	0.93	0.3519
	Mean age in cases, year	−0.0633	0.1114	−0.2815	0.1550	−0.57	0.5701
	Mean AHI in cases, events/h	0.0087	0.0462	−0.0818	0.0991	0.19	0.8512
	Mean BMI in controls, kg/m^2^	−0.0143	0.1951	−0.3967	0.3680	−0.07	0.9415
	Mean age in controls, year	0.0786	0.0731	−0.0647	0.2220	1.08	0.2822
TNF-α (children)	Publication year	0.0006	0.0005	−0.0004	0.0017	1.25	0.2104
	Sample size	−0.0080	0.0095	−0.0267	0.0107	−0.84	0.4015
	Mean BMI in cases, kg/m^2^	0.0196	0.0128	−0.0056	0.0448	1.53	0.1267
	Mean age in cases, year	0.0462	0.0309	−0.0142	0.1067	1.50	0.1340
	Mean AHI in cases, events/h	0.0524	0.0244	0.0047	0.1002	2.15	**0.0314**
	Mean BMI in controls, kg/m^2^	0.0229	0.0140	−0.0045	0.0503	1.64	0.1011
	Mean age in controls, year	0.0466	0.0302	−0.0125	0.1057	1.54	0.1224
IL-8 (children)	Publication year	0.0003	0.0001	0.0002	0.0004	4.90	**<0.0001**
	Sample size	0.0046	0.0010	0.0028	0.0065	4.80	**<0.0001**
	Mean BMI in cases, kg/m^2^	0.0263	0.0054	0.0157	0.0368	4.89	**<0.0001**
	Mean age in cases, year	0.0645	0.0131	0.0388	0.0902	4.91	**<0.0001**
	Mean AHI in cases, events/h	0.0574	0.0119	0.0340	0.0808	4.80	**<0.0001**
	Mean BMI in controls, kg/m^2^	0.0289	0.0059	0.0174	0.0404	4.92	**<0.0001**
	Mean age in controls, year	0.0605	0.0123	0.0364	0.0845	4.92	**<0.0001**

Bold number means statistically significant (*p* < 0.05).

**Table 5:** Publication bias analysis.

Biomarker	Egger’s Test, *p*-Value	Begg’s Test, *p*-Value
TNF-α (adult)	**<0.0001**	**<0.0001**
IL-8 (adult)	0.3924	0.3304
IL-1β (adult)	0.7117	0.9698
IFN-γ (adult)	0.1884	0.1416
TNF-α (children)	0.2183	0.2799
IL-8 (children)	**0.0507**	0.1764
IL-1β (children)	0.4726	0.3272

Bold number means statistically significant (*p* < 0.10).

## Data Availability

All data obtained were included in this article.

## Supplementary Materials

The following supporting information can be downloaded at: https://www.mdpi.com/article/10.3390/jcm13051484/s1, Supplementary File S1: Quality score of the articles; Supplementary File S2: Funnel plot analysis of comparison of serum/plasma levels of cytokines; Supplementary File S3: Trial sequential plot analysis of comparison of serum/plasma levels of cytokines.

## References

[1] Senaratna C.V., Perret J.L., Lodge C.J., Lowe A.J., Campbell B.E., Matheson M.C., Hamilton G.S., Dharmage S.C. (2017). Prevalence of obstructive sleep apnea in the general population: A systematic review. Sleep Med. Rev..

[2] Heinzer R., Vat S., Marques-Vidal P., Marti-Soler H., Andries D., Tobback N., Mooser V., Preisig M., Malhotra A., Waeber G. (2015). Prevalence of sleep-disordered breathing in the general population: The HypnoLaus study. Lancet Respir. Med..

[3] Benjafield A.V., Ayas N.T., Eastwood P.R., Heinzer R., Ip M.S., Morrell M.J., Nunez C.M., Patel S.R., Penzel T., Pépin J.-L. (2019). Estimation of the global prevalence and burden of obstructive sleep apnoea: A literature-based analysis. Lancet Respir. Med..

[4] Kapur V.K., Auckley D.H., Chowdhuri S., Kuhlmann D.C., Mehra R., Ramar K., Harrod C.G. (2017). Clinical practice guideline for diagnostic testing for adult obstructive sleep apnea: An American Academy of Sleep Medicine clinical practice guideline. J. Clin. Sleep Med..

[5] Semelka M., Wilson J., Floyd R. (2016). Diagnosis and treatment of obstructive sleep apnea in adults. Am. Fam. Physician.

[6] Young T., Palta M., Dempsey J., Peppard P.E., Nieto F.J., Hla K.M. (2009). Burden of sleep apnea: Rationale, design, and major findings of the Wisconsin Sleep Cohort study. WMJ Off. Publ. State Med. Soc. Wis..

[7] Peppard P.E., Young T., Barnet J.H., Palta M., Hagen E.W., Hla K.M. (2013). Increased prevalence of sleep-disordered breathing in adults. Am. J. Epidemiol..

[8] Gamsiz-Isik H., Kiyan E., Bingol Z., Baser U., Ademoglu E., Yalcin F. (2017). Does obstructive sleep apnea increase the risk for periodontal disease? A case-control study. J. Periodontol..

[9] Dempsey J.A., Veasey S.C., Morgan B.J., O’Donnell C.P. (2010). Pathophysiology of sleep apnea. Physiol. Rev..

[10] Bixler E.O., Vgontzas A.N., Lin H.-M., Liao D., Calhoun S., Vela-Bueno A., Fedok F., Vlasic V., Graff G. (2009). Sleep disordered breathing in children in a general population sample: Prevalence and risk factors. Sleep.

[11] Kang M., Mo F., Witmans M., Santiago V., Tablizo M.A. (2022). Trends in diagnosing obstructive sleep apnea in pediatrics. Children.

[12] Bhatt S.P., Guleria R., Kabra S.K. (2021). Metabolic alterations and systemic inflammation in overweight/obese children with obstructive sleep apnea. PLoS ONE.

[13] Hirsch D., Evans C.A., Wong M., Machaalani R., Waters K.A. (2019). Biochemical markers of cardiac dysfunction in children with obstructive sleep apnoea (OSA). Sleep Breath..

[14] Boyd J.H., Petrof B.J., Hamid Q., Fraser R., Kimoff R.J. (2004). Upper airway muscle inflammation and denervation changes in obstructive sleep apnea. Am. J. Respir. Crit. Care Med..

[15] Paulsen F.P., Steven P., Tsokos M., Jungmann K., Müller A., Verse T., Pirsig W. (2002). Upper airway epithelial structural changes in obstructive sleep-disordered breathing. Am. J. Respir. Crit. Care Med..

[16] Vicente E., Marin J.M., Carrizo S.J., Osuna C.S., González R., Marin-Oto M., Forner M., Vicente P., Cubero P., Gil A.V. (2016). Upper airway and systemic inflammation in obstructive sleep apnoea. Eur. Respir. J..

[17] Imani M.M., Sadeghi M., Farokhzadeh F., Khazaie H., Brand S., Dürsteler K.M., Brühl A., Sadeghi-Bahmani D. (2021). Evaluation of Blood Levels of C-Reactive Protein Marker in Obstructive Sleep Apnea: A Systematic Review, Meta-Analysis and Meta-Regression. Life.

[18] Imani M.M., Sadeghi M., Gholamipour M.A., Brühl A.B., Sadeghi-Bahmani D., Brand S. (2022). Evaluation of Blood Intercellular Adhesion Molecule-1 (ICAM-1) Level in Obstructive Sleep Apnea: A Systematic Review and Meta-Analysis. Medicina.

[19] Imani M.M., Sadeghi M., Khazaie H., Emami M., Bahmani D.S., Brand S. (2020). Evaluation of serum and plasma interleukin-6 levels in obstructive sleep apnea syndrome: A meta-analysis and meta-regression. Front. Immunol..

[20] Imani M.M., Sadeghi M., Khazaie H., Emami M., Bahmani D.S., Brand S. (2020). Serum and plasma tumor necrosis factor alpha levels in individuals with obstructive sleep apnea syndrome: A meta-analysis and meta-regression. Life.

[21] Doğan F.Ü., Yosunkaya Ş., Okur H.K., Can Ü. (2014). Relationships between obstructive sleep apnea syndrome, continuous positive airway pressure treatment, and inflammatory cytokines. Sleep Disord..

[22] Kapsimalis F., Richardson G., Opp M.R., Kryger M. (2005). Cytokines and normal sleep. Curr. Opin. Pulm. Med..

[23] Testelmans D., Tamisier R., Barone-Rochette G., Baguet J.-P., Roux-Lombard P., Pépin J.-L., Lévy P. (2013). Profile of circulating cytokines: Impact of OSA, obesity and acute cardiovascular events. Cytokine.

[24] Li Q., Zheng X. (2017). Tumor necrosis factor alpha is a promising circulating biomarker for the development of obstructive sleep apnea syndrome: A meta-analysis. Oncotarget.

[25] Yi M., Zhao W., Tan Y., Fei Q., Liu K., Chen Z., Zhang Y. (2022). The causal relationships between obstructive sleep apnea and elevated CRP and TNF-α protein levels. Ann. Med..

[26] Cao Y., Song Y., Ning P., Zhang L., Wu S., Quan J., Li Q. (2020). Association between tumor necrosis factor alpha and obstructive sleep apnea in adults: A meta-analysis update. BMC Pulm. Med..

[27] Zeng Q.-C., Sun Q., Zhang M., Tang Y., Long H.-C. (2021). Relation between IL-8 level and obstructive sleep apnea syndrome. Open Med..

[28] Janmohammadi P., Raeisi T., Zarei M., Nejad M.M., Karimi R., Mirali Z., Zafary R., Alizadeh S. (2023). Adipocytokines in obstructive sleep apnea: A systematic review and meta-analysis. Respir. Med..

[29] Nadeem R., Molnar J., Madbouly E.M., Nida M., Aggarwal S., Sajid H., Naseem J., Loomba R. (2013). Serum inflammatory markers in obstructive sleep apnea: A meta-analysis. J. Clin. Sleep Med..

[30] Li X., Hu R., Ren X., He J. (2021). Interleukin-8 concentrations in obstructive sleep apnea syndrome: A systematic review and meta-analysis. Bioengineered.

[31] Akinnusi M., Jaoude P., Kufel T., El-Solh A.A. (2013). Toll-like receptor activity in patients with obstructive sleep apnea. Sleep Breath..

[32] Bhushan B., Guleria R., Misra A., Luthra K., Vikram N.K. (2009). TNF-alpha gene polymorphism and TNF-alpha levels in obese Asian Indians with obstructive sleep apnea. Respir. Med..

[33] Ciftci T.U., Kokturk O., Bukan N., Bilgihan A. (2004). The relationship between serum cytokine levels with obesity and obstructive sleep apnea syndrome. Cytokine.

[34] Feng Y., Ma L., Chen X., Zhang Y., Cao Z., Yuan Y., Xie Y., Liu H., Shi Y., Ren X. (2022). Relationship between serum brain-derived neurotrophic factor and cognitive impairment in children with sleep-disordered breathing. Front. Pediatr..

[35] Huang Y.S., Guilleminault C., Hwang F.M., Cheng C., Lin C.H., Li H.Y., Lee L.A. (2016). Inflammatory cytokines in pediatric obstructive sleep apnea. Medicine.

[36] Epstein L.J., Kristo D., Strollo P.J., Friedman N., Malhotra A., Patil S.P., Ramar K., Rogers R., Schwab R.J., Weaver E.M. (2009). Clinical guideline for the evaluation, management and long-term care of obstructive sleep apnea in adults. J. Clin. Sleep Med..

[37] Farber J.M. (2002). Clinical practice guideline: Diagnosis and management of childhood obstructive sleep apnea syndrome. Pediatrics.

[38] Stang A. (2010). Critical evaluation of the Newcastle-Ottawa scale for the assessment of the quality of nonrandomized studies in meta-analyses. Eur. J. Epidemiol..

[39] DerSimonian R., Laird N. (2015). Meta-analysis in clinical trials revisited. Contemp. Clin. Trials.

[40] Mantel N., Haenszel W. (1959). Statistical aspects of the analysis of data from retrospective studies of disease. J. Natl. Cancer Inst..

[41] Wetterslev J., Jakobsen J.C., Gluud C. (2017). Trial sequential analysis in systematic reviews with meta-analysis. BMC Med. Res. Methodol..

[42] Abdel-Fadeil M.R., Abedelhaffez A.S., Makhlouf H.A., Al Qirshi G.A. (2017). Obstructive sleep apnea: Influence of hypertension on adiponectin, inflammatory markers and dyslipidemia. Pathophysiology.

[43] Abulikemu Y., Abulajang T., Ailigen A., Tang L. (2021). Analysis of propensity score matching between inflammatory factor levels and gene polymorphisms and susceptibility to obstructive sleep apnea. Lin Chuang Er Bi Yan Hou Tou Jing Wai Ke Za Zhi = J. Clin. Otorhinolaryngol. Head Neck Surg..

[44] Ahsant S., Rahmani Fard S., Riahi T., Taheri Tinjani R., Shamlou Mahmoudi F., Alimohamadi Y., Kooranifar S., Hosseinipour A., Minaeian S. (2022). Evaluation the Possible Role of Interleukin-6 and Tumor Necrosis Factor-Alpha in Pathogenesis of Obstructive Sleep Apnea in Obese Patients: A Case-Control Study. Galen Med. J..

[45] Alzoghaibi M.A., Bahammam A.S. (2005). Lipid peroxides, superoxide dismutase and circulating IL-8 and GCP-2 in patients with severe obstructive sleep apnea: A pilot study. Sleep Breath..

[46] Archontogeorgis K., Nena E., Tsigalou C., Voulgaris A., Xanthoudaki M., Froudarakis M., Steiropoulos P. (2016). Cystatin C levels in middle-aged patients with obstructive sleep apnea syndrome. Pulm. Med..

[47] Arias M., García-Río F., Alonso-Fernández A., Hernanz A., Hidalgo R., Martínez-Mateo V., Bartolomé S., Rodríguez-Padial L. (2008). CPAP decreases plasma levels of soluble tumour necrosis factor-α receptor 1 in obstructive sleep apnoea. Eur. Respir. J..

[48] Bhatt S.P., Guleria R., Vikram N.K., Gupta A.K. (2019). Non-alcoholic fatty liver disease is an independent risk factor for inflammation in obstructive sleep apnea syndrome in obese Asian Indians. Sleep Breath..

[49] Bilal N., Kurutas E.B., Orhan I., Bilal B., Doganer A. (2021). Evaluation of preoperative and postoperative serum interleukin-6, interleukin-8, tumor necrosis factor α and raftlin levels in patients with obstructive sleep apnea. Sleep Breath..

[50] Bozic J., Borovac J.A., Galic T., Kurir T.T., Supe-Domic D., Dogas Z. (2018). Adropin and inflammation biomarker levels in male patients with obstructive sleep apnea: A link with glucose metabolism and sleep parameters. J. Clin. Sleep Med..

[51] Carneiro G., Togeiro S.M., Ribeiro-Filho F.F., Truksinas E., Ribeiro A.B., Zanella M.T., Tufik S. (2009). Continuous positive airway pressure therapy improves hypoadiponectinemia in severe obese men with obstructive sleep apnea without changes in insulin resistance. Metab. Syndr. Relat. Disord..

[52] Carpagnano G.E., Spanevello A., Sabato R., Depalo A., Palladino G.P., Bergantino L., Barbaro M.P.F. (2010). Systemic and airway inflammation in sleep apnea and obesity: The role of ICAM-1 and IL-8. Transl. Res..

[53] Celikhisar H., Ilkhan G.D. (2020). Alterations in serum adropin, adiponectin, and proinflammatory cytokine levels in OSAS. Can. Respir. J..

[54] Chen B., Liu Y.N., Ji L., Liu P.L., He J., Gan Y.Y., Ji G.J., Zhu S.Y., Zhang W.H. (2021). Elevated levels of interleukin-35 and interleukin-37 in adult patients with obstructive sleep apnea. J. Clin. Lab. Anal..

[55] Chen B., Zhang W., Chen Y., Hu C., Bian H., He J., Ji L., Zhu S. (2015). Association of obstructive sleep apnea hypopnea syndrome with carotid atherosclerosis and the efficacy of continuous positive airway pressure treatment. Zhonghua Yi Xue Za Zhi.

[56] Chen P.C., Guo C.H., Tseng C.J., Wang K.C., Liu P.J. (2013). Blood trace minerals concentrations and oxidative stress in patients with obstructive sleep apnea. J. Nutr. Health Aging.

[57] Ciccone M.M., Scicchitano P., Zito A., Cortese F., Boninfante B., Falcone V.A., Quaranta V.N., Ventura V.A., Zucano A., Di Serio F. (2014). Correlation between inflammatory markers of atherosclerosis and carotid intima-media thickness in obstructive sleep apnea. Molecules.

[58] Constantinidis J., Ereliadis S., Angouridakis N., Konstantinidis I., Vital V., Angouridaki C. (2008). Cytokine changes after surgical treatment of obstructive sleep apnoea syndrome. Eur. Arch. Oto-Rhino-Laryngol..

[59] De Santis S., Cambi J., Tatti P., Bellussi L., Passali D. (2015). Changes in ghrelin, leptin and pro-inflammatory cytokines after therapy in Obstructive Sleep Apnea Syndrome (OSAS) patients. Otolaryngol. Pol..

[60] Devouassoux G., Lévy P., Rossini E., Pin I., Fior-Gozlan M., Henry M., Seigneurin D., Pépin J.-L. (2007). Sleep apnea is associated with bronchial inflammation and continuous positive airway pressure–induced airway hyperresponsiveness. J. Allergy Clin. Immunol..

[61] Doufas A.G., Tian L., Padrez K.A., Suwanprathes P., Cardell J.A., Maecker H.T., Panousis P. (2013). Experimental pain and opioid analgesia in volunteers at high risk for obstructive sleep apnea. PLoS ONE.

[62] Fiedorczuk P., Olszewska E., Polecka A., Walasek M., Mroczko B., Kulczyńska-Przybik A. (2023). Investigating the Role of Serum and Plasma IL-6, IL-8, IL-10, TNF-alpha, CRP, and S100B Concentrations in Obstructive Sleep Apnea Diagnosis. Int. J. Mol. Sci..

[63] Fornadi K., Lindner A., Czira M.E., Szentkiralyi A., Lazar A.S., Zoller R., Turanyi C.Z., Veber O., Novak M., Mucsi I. (2012). Lack of association between objectively assessed sleep disorders and inflammatory markers among kidney transplant recipients. Int. Urol. Nephrol..

[64] Galati D., Zanotta S., Canora A., Polistina G.E., Nicoletta C., Ghinassi G., Ciasullo E., Bocchino M. (2020). Severe depletion of peripheral blood dendritic cell subsets in obstructive sleep apnea patients: A new link with cancer?. Cytokine.

[65] Bao H.R., Yu Q., Liu X.J., Wang X.Y. (2005). Changes of serum interleukin-8 and monocyte chemoattractant protein-1 levels in patients with obstructive sleep apnea hypopnea syndrome. Chin. J. Clin. Rehabil..

[66] Hargens T.A., Guill S.G., Kaleth A.S., Nickols-Richardson S.M., Miller L.E., Zedalis D., Gregg J.M., Gwazdauskas F., Herbert W.G. (2013). Insulin resistance and adipose-derived hormones in young men with untreated obstructive sleep apnea. Sleep Breath..

[67] Heizati M., Li N., Shao L., Yao X., Wang Y., Hong J., Zhou L., Zhang D., Chang G., Abulikemu S. (2017). Does increased serum d-lactate mean subclinical hyperpermeability of intestinal barrier in middle-aged nonobese males with OSA?. Medicine.

[68] Hirotsu C., Albuquerque R.G., Nogueira H., Hachul H., Bittencourt L., Tufik S., Andersen M.L. (2017). The relationship between sleep apnea, metabolic dysfunction and inflammation: The gender influence. Brain Behav. Immun..

[69] Hui P., Jia S., Ma W., Zhao L., Wang J., Wei X., Zhou L., Dai M., Zhang W., Xie Y. (2016). The clinical significance and changes of serum tumor necrosis factor and plasma endothelium in patients with OSAHS associated Type 2 diabetes mellites. Lin Chuang Er Bi Yan Hou Tou Jing Wai Ke Za Zhi.

[70] Huiguo L., Jin L., Shengdao X., Guanxin S., Zhenxiang Z., Yongjian X. (2000). The change of interleukin-6 and tumor necrosis factor in patients with obstructive sleep apnea syndrome. J. Tongji Med. Univ..

[71] Imagawa S., Yamaguchi Y., Ogawa K., Obara N., Suzuki N., Yamamoto M., Nagasawa T. (2004). Interleukin-6 and tumor necrosis factor-alpha in patients with obstructive sleep apnea-hypopnea syndrome. Respiration.

[72] Ji L., Liu Y., Liu P., Ji G., He J., Gan Y., Zhu S., Chen B., Zhang W. (2021). Serum periostin and TNF-α levels in patients with obstructive sleep apnea-hypopnea syndrome. Sleep Breath..

[73] Ji P., Kou Q., Zhang J. (2022). Study on relationship between carotid intima-media thickness and inflammatory factors in obstructive sleep apnea. Nat. Sci. Sleep.

[74] Jiang Y.Q., Xue J.S., Xu J., Zhou Z.X., Ji Y.L. (2017). Efficacy of continuous positive airway pressure treatment in treating obstructive sleep apnea hypopnea syndrome associated with carotid arteriosclerosis. Exp. Ther. Med..

[75] Jin F., Liu J., Zhang X., Cai W., Zhang Y., Zhang W., Yang J., Lu G., Zhang X. (2017). Effect of continuous positive airway pressure therapy on inflammatory cytokines and atherosclerosis in patients with obstructive sleep apnea syndrome. Mol. Med. Rep..

[76] Kanbay A., Kokturk O., Ciftci T.U., Tavil Y., Bukan N. (2008). Comparison of serum adiponectin and tumor necrosis factor-alpha levels between patients with and without obstructive sleep apnea syndrome. Respiration.

[77] Kim J., Lee C.H., Park C.S., Kim B.G., Kim S.W., Cho J.H. (2010). Plasma levels of MCP-1 and adiponectin in obstructive sleep apnea syndrome. Arch. Otolaryngol. Neck Surg..

[78] Ko C.Y., Hu A.K., Chou D., Huang L.M., Su H.Z., Yan F.R., Zhang X.B., Zhang H.P., Zeng Y.M. (2019). Analysis of oral microbiota in patients with obstructive sleep apnea-associated hypertension. Hypertens. Res..

[79] Kobayashi K., Nishimura Y., Shimada T., Yoshimura S., Funada Y., Satouchi M., Yokoyama M. (2006). Effect of continuous positive airway pressure on soluble CD40 ligand in patients with obstructive sleep apnea syndrome. Chest.

[80] Kong Y., Li Z., Tang T., Wu H., Liu J., Gu L., Zhao T., Huang Q. (2018). The level of lipopolysaccharide-binding protein is elevated in adult patients with obstructive sleep apnea. BMC Pulm. Med..

[81] Leon-Cabrera S., Arana-Lechuga Y., Esqueda-León E., Terán-Pérez G., Gonzalez-Chavez A., Escobedo G., Moctezuma J.V. (2015). Reduced systemic levels of IL-10 are associated with the severity of obstructive sleep apnea and insulin resistance in morbidly obese humans. Mediat. Inflamm..

[82] Li X., Liu X., Meng Q., Wu X., Bing X., Guo N., Zhao X., Hou X., Wang B., Xia M. (2022). Circadian clock disruptions link oxidative stress and systemic inflammation to metabolic syndrome in obstructive sleep apnea patients. Front. Physiol..

[83] Li Y., Chongsuvivatwong V., Geater A., Liu A. (2008). Are biomarker levels a good follow-up tool for evaluating obstructive sleep apnea syndrome treatments?. Respiration.

[84] Li Y., Chongsuvivatwong V., Geater A., Liu A. (2009). Exhaled breath condensate cytokine level as a diagnostic tool for obstructive sleep apnea syndrome. Sleep Med..

[85] Lin C.C., Liaw S.F., Chiu C.H., Chen W.J., Lin M.W., Chang F.T. (2016). Effects of nasal CPAP on exhaled SIRT1 and tumor necrosis factor-α in patients with obstructive sleep apnea. Respir. Physiol. Neurobiol..

[86] Lu D., Xu S., Dai P., Wu L., Zhang H., Zhou B. (2022). Gut microbiota in hypertensive patients with versus without obstructive sleep apnea. J. Clin. Hypertens..

[87] Matos G., Hirotsu C., Alvarenga T.A., Cintra F., Bittencourt L., Tufik S., Andersen M.L. (2013). The association between TNF-α and erectile dysfunction complaints. Andrology.

[88] Medeiros C., De Bruin V., Andrade G., Coutinho W., de Castro-Silva C., De Bruin P. (2012). Obstructive sleep apnea and biomarkers of inflammation in ischemic stroke. Acta Neurol. Scand..

[89] Ming H., Tian A., Liu B., Hu Y., Liu C., Chen R., Cheng L. (2019). Inflammatory cytokines tumor necrosis factor-α, interleukin-8 and sleep monitoring in patients with obstructive sleep apnea syndrome. Exp. Ther. Med..

[90] Minoguchi K., Tazaki T., Yokoe T., Minoguchi H., Watanabe Y., Yamamoto M., Adachi M. (2004). Elevated production of tumor necrosis factor-alpha by monocytes in patients with obstructive sleep apnea syndrome. Chest.

[91] Nizam N., Basoglu O.K., Tasbakan M.S., Lappin D.F., Buduneli N. (2016). Is there an association between obstructive sleep apnea syndrome and periodontal inflammation?. Clin. Oral Investig..

[92] Niżankowska-Jędrzejczyk A., Almeida F.R., Lowe A.A., Kania A., Nastałek P., Mejza F., Foley J.H., Niżankowska-Mogilnicka E., Undas A. (2014). Modulation of inflammatory and hemostatic markers in obstructive sleep apnea patients treated with mandibular advancement splints: A parallel, controlled trial. J. Clin. Sleep Med..

[93] Ohga E., Tomita T., Wada H., Yamamoto H., Nagase T., Ouchi Y. (2003). Effects of obstructive sleep apnea on circulating ICAM-1, IL-8, and MCP-1. J. Appl. Physiol..

[94] Olszewska E., Pietrewicz T.M., Świderska M., Jamiołkowski J., Chabowski A. (2022). A Case-Control Study on the Changes in High-Sensitivity C-Reactive Protein and Tumor Necrosis Factor-Alpha Levels with Surgical Treatment of OSAS. Int. J. Mol. Sci..

[95] Qian X., Yin T., Li T., Kang C., Guo R., Sun B., Liu C. (2012). High levels of inflammation and insulin resistance in obstructive sleep apnea patients with hypertension. Inflammation.

[96] Ryan S., Taylor C.T., McNicholas W.T. (2005). Selective activation of inflammatory pathways by intermittent hypoxia in obstructive sleep apnea syndrome. Circulation.

[97] Ryan S., Taylor C.T., McNicholas W.T. (2006). Predictors of elevated nuclear factor-kappaB-dependent genes in obstructive sleep apnea syndrome. Am. J. Respir. Crit. Care Med..

[98] Sahlman J., Miettinen K., Peuhkurinen K., Seppä J., Peltonen M., Herder C., Punnonen K., Vanninen E., Gylling H., Partinen M. (2010). The activation of the inflammatory cytokines in overweight patients with mild obstructive sleep apnoea. J. Sleep Res..

[99] Said E.A., Al-Abri M.A., Al-Saidi I., Al-Balushi M.S., Al-Busaidi J.Z., Al-Reesi I., Koh C.Y., Hasson S.S., Idris M.A., Al-Jabri A.A. (2017). Altered blood cytokines, CD4 T cells, NK and neutrophils in patients with obstructive sleep apnea. Immunol. Lett..

[100] Santamaria-Martos F., Benítez I., Girón C., Barbé F., Martínez-García M.A., Hernández L., Montserrat J.M., Nagore E., Martorell A., Campos-Rodriguez F. (2018). Biomarkers of carcinogenesis and tumour growth in patients with cutaneous melanoma and obstructive sleep apnoea. Eur. Respir. J..

[101] Sarac F., Basoglu O.K., Gunduz C., Bayrak H., Biray Avci C., Akcicek F. (2011). Association of osteopontin and tumor necrosis factor-α levels with insulin resistance in obese patients with obstructive sleep apnea syndrome. J. Endocrinol. Investig..

[102] Ulasli S.S., Sarıaydın M., Gunay E., Halici B., Celik S., Koyuncu T., Ulu S., Unlu M. (2015). Effects of nondipping pattern on systemic inflammation in obstructive sleep apnea. Sleep Breath..

[103] Serednytskyy O., Alonso-Fernández A., Ribot C., Herranz A., Álvarez A., Sánchez A., Rodríguez P., Gil A.V., Pía C., Cubero J.P. (2022). Systemic inflammation and sympathetic activation in gestational diabetes mellitus with obstructive sleep apnea. BMC Pulm. Med..

[104] Sun L., Chen R., Wang J., Zhang Y., Li J., Peng W., Liu C. (2014). Association between inflammation and cognitive function and effects of continuous positive airway pressure treatment in obstructive sleep apnea hypopnea syndrome. Zhonghua Yi Xue Za Zhi.

[105] Tamaki S., Yamauchi M., Fukuoka A., Makinodan K., Koyama N., Tomoda K., Yoshikawa M., Kimura H. (2009). Production of inflammatory mediators by monocytes in patients with obstructive sleep apnea syndrome. Intern. Med..

[106] Tang T., Huang Q., Liu J., Zhou X., Du J., Wu H., Li Z. (2019). Oxidative stress does not contribute to the release of proinflammatory cytokines through activating the Nod-like receptor protein 3 inflammasome in patients with obstructive sleep apnoea. Sleep Breath..

[107] Tazaki T., Minoguchi K., Yokoe T., Samson K.T., Minoguchi H., Tanaka A., Watanabe Y., Adachi M. (2004). Increased levels and activity of matrix metalloproteinase-9 in obstructive sleep apnea syndrome. Am. J. Respir. Crit. Care Med..

[108] Thorn C.E., Knight B., Pastel E., McCulloch L., Patel B., Shore A., Kos K. (2017). Adipose tissue is influenced by hypoxia of obstructive sleep apnea syndrome independent of obesity. Diabetes Metab..

[109] Thunström E., Glantz H., Fu M., Yucel-Lindberg T., Petzold M., Lindberg K., Peker Y. (2015). Increased inflammatory activity in nonobese patients with coronary artery disease and obstructive sleep apnea. Sleep.

[110] Tomiyama H., Okazaki R., Inoue D., Ochiai H., Shiina K., Takata Y., Hashimoto H., Yamashina A. (2008). Link between obstructive sleep apnea and increased bone resorption in men. Osteoporos. Int..

[111] Tosun F., Babayiğit C., Dikmen N., Doğan S., Dirican E. (2023). The effect of continuous positive airway pressure treatment on inflammatory parameters and periostin levels in patients with obstructive sleep apnea syndrome. Sleep Breath..

[112] Vgontzas A.N., Papanicolaou D.A., Bixler E.O., Hopper K., Lotsikas A., Lin H.M., Kales A., Chrousos G.P. (2000). Sleep apnea and daytime sleepiness and fatigue: Relation to visceral obesity, insulin resistance, and hypercytokinemia. J. Clin. Endocrinol. Metab..

[113] Vgontzas A.N., Papanicolaou D.A., Bixler E.O., Kales A., Tyson K., Chrousos G.P. (1997). Elevation of plasma cytokines in disorders of excessive daytime sleepiness: Role of sleep disturbance and obesity. J. Clin. Endocrinol. Metab..

[114] Wali S.O., Manzar M.D., Abdelaziz M.M., Alshomrani R., Alhejaili F., Al-Mughales J., Alamoudi W., Gozal D. (2021). Putative associations between inflammatory biomarkers, obesity, and obstructive sleep apnea. Ann. Thorac. Med..

[115] Wang J., Li X., Hou W.J., Dong L.X., Cao J. (2019). Endothelial function and T-lymphocyte subsets in patients with overlap syndrome of chronic obstructive pulmonary disease and obstructive sleep apnea. Chin. Med. J..

[116] Xie J.Y., Liu W.X., Ji L., Chen Z., Gao J.M., Chen W., Chen G.F., Zhu Q. (2020). Relationship between inflammatory factors and arrhythmia and heart rate variability in OSAS patients. Eur. Rev. Med. Pharmacol. Sci..

[117] Yadav R., France M., Aghamohammadzadeh R., Liu Y., Hama S., Kwok S., Schofield J., Turkington P., Syed A.A., Malik R. (2014). Impairment of high-density lipoprotein resistance to lipid peroxidation and adipose tissue inflammation in obesity complicated by obstructive sleep apnea. J. Clin. Endocrinol. Metab..

[118] Yang D., Liu Z., Luo Q. (2013). Plasma ghrelin and pro-inflammatory markers in patients with obstructive sleep apnea and stable coronary heart disease. Med. Sci. Monit..

[119] Yang Y., Somani S. (2023). Impact of obstructive sleep apnea on the expression of inflammatory mediators in diabetic macular edema. Eur. J. Ophthalmol..

[120] Zong D., Liu X., Shen C., Liu T., Ouyang R. (2023). Involvement of Galectin-3 in neurocognitive impairment in obstructive sleep apnea via regulating inflammation and oxidative stress through NLRP3. Sleep Med..

[121] Gaines J., Vgontzas A.N., Fernandez-Mendoza J., Calhoun S.L., He F., Liao D., Sawyer M.D., Bixler E.O. (2016). Inflammation mediates the association between visceral adiposity and obstructive sleep apnea in adolescents. Am. J. Physiol. Endocrinol. Metab..

[122] Huang Y.S., Chin W.C., Guilleminault C., Chu K.C., Lin C.H., Li H.Y. (2020). Inflammatory Factors: Nonobese Pediatric Obstructive Sleep Apnea and Adenotonsillectomy. J. Clin. Med..

[123] Jie C., Jun Y., Wen-wei Y., Hao W. (2007). Analysis of Serum IL-6 and TNF-α Levels in Children with Obstructive Sleep Apnea Syndrome. Chin. J. Evid.-Based Med..

[124] Khalyfa A., Serpero L.D., Kheirandish-Gozal L., Capdevila O.S., Gozal D. (2011). TNF-α gene polymorphisms and excessive daytime sleepiness in pediatric obstructive sleep apnea. J. Pediatr..

[125] Li A.M., Lam H.S., Chan M., So H.K., Ng S.K., Chan I., Lam C., Wing Y.K. (2008). Inflammatory cytokines and childhood obstructive sleep apnoea. Ann. Acad. Med. Singap..

[126] Li J., Li C., Chai L., Gong W. (2014). Relationship between plasma vascular endothelial growth factor and tumor necrosis factor-alpha and obstructive sleep apnea hypopnea syndrome in children. Zhonghua Er Bi Yan Hou Tou Jing Wai Ke Za Zhi.

[127] Nobili V., Alisi A., Cutrera R., Carpino G., De Stefanis C., D’Oria V., De Vito R., Cucchiara S., Gaudio E., Musso G. (2015). Altered gut–liver axis and hepatic adiponectin expression in OSAS: Novel mediators of liver injury in paediatric non-alcoholic fatty liver. Thorax.

[128] Smith D.F., Hossain M.M., Hura A., Huang G., McConnell K., Ishman S.L., Amin R.S. (2017). Inflammatory Milieu and Cardiovascular Homeostasis in Children with Obstructive Sleep Apnea. Sleep.

[129] Smith D.F., Schuler C.L., Hossain M.M., Huang G., McConnell K., Urbina E.M., Amin R.S. (2021). Early Atherosclerotic inflammatory pathways in children with obstructive sleep apnea. J. Pediatr..

[130] Tam C.S., Wong M., McBain R., Bailey S., Waters K.A. (2006). Inflammatory measures in children with obstructive sleep apnoea. J. Paediatr. Child Health.

[131] Wang Y., Chen Y., Lin W., Huang M., Xu Y., Chen G. (2023). Inflammatory markers in children with obstructive sleep apnea syndrome. Front. Pediatr..

[132] Ye J., Liu H., Li P., Chen Z.G., Zhang G.H., Yang Q.T., Li Y. (2015). CD4^+^T-lymphocyte subsets in nonobese children with obstructive sleep apnea syndrome. Pediatr. Res..

[133] Zhang Z., Wang C. (2017). Immune status of children with obstructive sleep apnea/hypopnea syndrome. Pak. J. Med. Sci..

[134] De Lima F.F.F., Mazzotti D.R., Tufik S., Bittencourt L. (2016). The role inflammatory response genes in obstructive sleep apnea syndrome: A review. Sleep Breath..

[135] Unnikrishnan D., Jun J., Polotsky V. (2015). Inflammation in sleep apnea: An update. Rev. Endocr. Metab. Disord..

[136] Quercioli A., Mach F., Montecucco F. (2010). Inflammation accelerates atherosclerotic processes in obstructive sleep apnea syndrome (OSAS). Sleep Breath..

[137] Aihara K., Oga T., Chihara Y., Harada Y., Tanizawa K., Handa T., Hitomi T., Uno K., Mishima M., Chin K. (2013). Analysis of systemic and airway inflammation in obstructive sleep apnea. Sleep Breath..

[138] Kleisiaris C.F., Kritsotakis E.I., Daniil Z., Tzanakis N., Papaioannou A., Gourgoulianis K.I. (2014). The prevalence of obstructive sleep apnea-hypopnea syndrome-related symptoms and their relation to airflow limitation in an elderly population receiving home care. Int. J. Chronic Obstr. Pulm. Dis..

[139] Cavaillon J.-M. (2001). Pro-versus anti-inflammatory cytokines: Myth or reality. Cell. Mol. Biol..

[140] Gottlieb D.J., Punjabi N.M. (2020). Diagnosis and management of obstructive sleep apnea: A review. JAMA.

[141] Reale M., Velluto L., Di Nicola M., D’Angelo C., Costantini E., Marchioni M., Cerroni G., Guarnieri B. (2020). Cholinergic markers and cytokines in OSA patients. Int. J. Mol. Sci..

[142] Liu X., Ma Y., Ouyang R., Zeng Z., Zhan Z., Lu H., Cui Y., Dai Z., Luo L., He C. (2020). The relationship between inflammation and neurocognitive dysfunction in obstructive sleep apnea syndrome. J. Neuroinflamm..

