# Supplementary material for: Association of Tumor Necrosis Factor-Alpha, Interleukin-1β, Interleukin-8, and Interferon-γ with Obstructive Sleep Apnea in Both Children and Adults: A Meta-Analysis of 102 Articles
Source: J Clin Med. 2024 Mar 4;13(5):1484. doi: 10.3390/jcm13051484 (PMC10932105; doi:10.3390/jcm13051484)
Supplement: Supplementary file 1 [file jcm-13-01484-s001.zip › Supplementary File S1.pdf]

Table S1: Quality score of each article in adults

First author, publication year	Selection	Comparability	Outcome	Total Quality score
Abdel-Fadeil, 2017 [42]	****	-	***	7
Abulikemu, 2021 [43]	****	*	***	8
Ahsant, 2022 [44]	****	*	***	8
Akinnusi, 2013 [31]	****	*	***	8
Alzoghaibi, 2005 [45]	****	-	***	7
Archontogeorgis, 2016 [46]	****	*	***	8
Arias, 2008[47]	****	*	***	8
Bhatt, 2019[48]	****	-	***	7
Bhushan, 2009 [32]	****	*	***	8
Bilal, 2021 [49]	****	*	***	8
Bozic, 2018[50]	****	*	***	8
Carneiro, 2009[51]	****	-	***	7
Carpagnano, 2010 [52]	****	-	***	7
Celikhisar, 2020 [53]	****	*	***	8
Chen, 2013[56]	****	-	***	7
Chen, 2015[55]	****	*	***	8
Chen, 2021 [54]	****	*	***	8
Ciccone, 2014[57]	****	*	***	8
Ciftci, 2004[33]	****	*	***	8
Constantinidis, 2008 [58]	****	*	***	8
De Santis, 2015 [59]	****	*	***	8
Devouassoux, 2007[60]	****	-	***	7
Doufas, 2013[61]	****	*	***	8
Fiedorczuk, 2023 [62]	****	*	***	8
Fornadi, 2012[63]	****	*	***	8
Galati, 2020[64]	****	**	***	9
Gamsiz-Isik, 2017 [8]	****	-	***	7
Hai-rong, 2005 [65]	****	-	***	7
Hargens, 2013[66]	****	*	***	8
Heizati, 2017[67]	****	**	***	9
Hirotsu, 2017[68]	****	-	***	7
Hui, 2016[69]	****	-	***	7
Huiguo, 2000[70]	****	*	***	8

Imagawa, 2004[71]	****	-	***	7
Ji, 2021 [72]	****	*	***	8
Ji, 2022 [73]	****	*	***	8
Jiang, 2017 [74]	****	-	***	7
Jin, 2017[75]	****	*	***	8
Kanbay, 2008 [76]	****	*	***	8
Kim, 2010 [77]	****	-	***	7
Ko, 2019[78]	****	-	***	7
Kobayashi, 2006[79]	****	*	***	8
Kong, 2018[80]	****	*	***	8
Leon-Cabrera, 2015[81]	****	-	***	7
Li, 2008a [83]	****	*	***	8
Li, 2009 [84]	****	*	***	8
Li, 2022 [82]	****	**	***	9
Lin, 2016[85]	****	*	***	8
Lu, 2022 [86]	****	**	***	9
Matos, 2013[87]	****	*	***	8
Medeiros, 2012[88]	****	*	***	8
Ming, 2019[89]	****	-	***	7
Minoguchi, 2004 [90]	****	-	***	7
Nizam, 2016[91]	****	*	***	8
Niżankowska-Jędrzejczyk, 2014 [92]	****	**	***	9
Ohga, 2003 [93]	****	**	***	9
Olszewska, 2022 [94]	****	-	***	7
Qian, 2012[95]	****	*	***	8
Ryan, 2005[96]	****	*	***	8
Ryan, 2006[97]	****	*	***	8
Sahlman, 2010[98]	****	*	***	8
Said, 2017 [99]	****	-	***	7
Santamaria-Martos, 2018 [100]	****	-	***	7
Sarac, 2011[101]	****	-	***	7
Sarinc Ulasli, 2015 [102]	****	*	***	8
Serednytskyy, 2022 [103]	****	*	***	8
Sun, 2014[104]	****	*	***	8
Tamaki, 2009 [105]	****	-	***	7

Tang, 2019[106]	****	*	***	8
Tazaki, 2004[107]	****	*	***	8
Thorn, 2017 [108]	****	*	***	8
Thunström, 2015 [109]	****	**	***	9
Tomiyama, 2008 [110]	****	*	***	8
Tosun, 2023 [111]	****	-	***	7
Unuvar Dogan, 2014 [21]	****	*	***	8
Vgontzas, 1997[113]	***	-	***	6
Vgontzas, 2000[112]	****	*	***	8
Vicente, 2016[16]	****	*	***	8
Wali, 2021 [114]	****	-	***	7
Wang, 2019 [115]	****	*	***	8
Xie, 2020 [116]	****	-	***	7
Yadav, 2014[117]	****	*	***	8
Yang, 2013 [118]	****	*	***	8
Yang, 2023 [119]	****	*	***	8
Zong, 2023 [120]	****	**	***	9

Each one asterisk (one point for each answer) shows one score. Maximum points: Selection: 4 scores. Comparability: 2 scores. Outcome: 3 scores.

Table S2: Quality score of each article in children

First author, publication year	**Selection**	**Comparability**	**Outcome**	**Total Quality score**
Bhatt, 2021[12]				9
Feng, 2022 [34]				9
Gaines, 2016[121]				7
Hirsch, 2019[13]				7
Huang, 2016 [35]				7
Huang, 2020[122]				9
Jie, 2007[123]				6
Khalyfa, 2011[124]				8
Li, 2008b [125]				7
Li, 2014[126]				8
Nobili, 2015 [127]				9
Smith, 2017[128]				7
Smith, 2021[129]				8
Tam, 2006[130]				9
Wang, 2023 [131]				9
Ye, 2015 [132]				7
Zhang, 2017 [133]				7

Each one asterisk ((one point for each answer)) shows one score. **Maximum points**: Selection: 4 scores. Comparability: 2 scores. Outcome: 3 scores.
