# Supplementary material for: Association of Tumor Necrosis Factor-Alpha, Interleukin-1β, Interleukin-8, and Interferon-γ with Obstructive Sleep Apnea in Both Children and Adults: A Meta-Analysis of 102 Articles
Source: J Clin Med. 2024 Mar 4;13(5):1484. doi: 10.3390/jcm13051484 (PMC10932105; doi:10.3390/jcm13051484)
Supplement: Supplementary file 1 [file jcm-13-01484-s001.zip › Supplementary File S3.pdf]

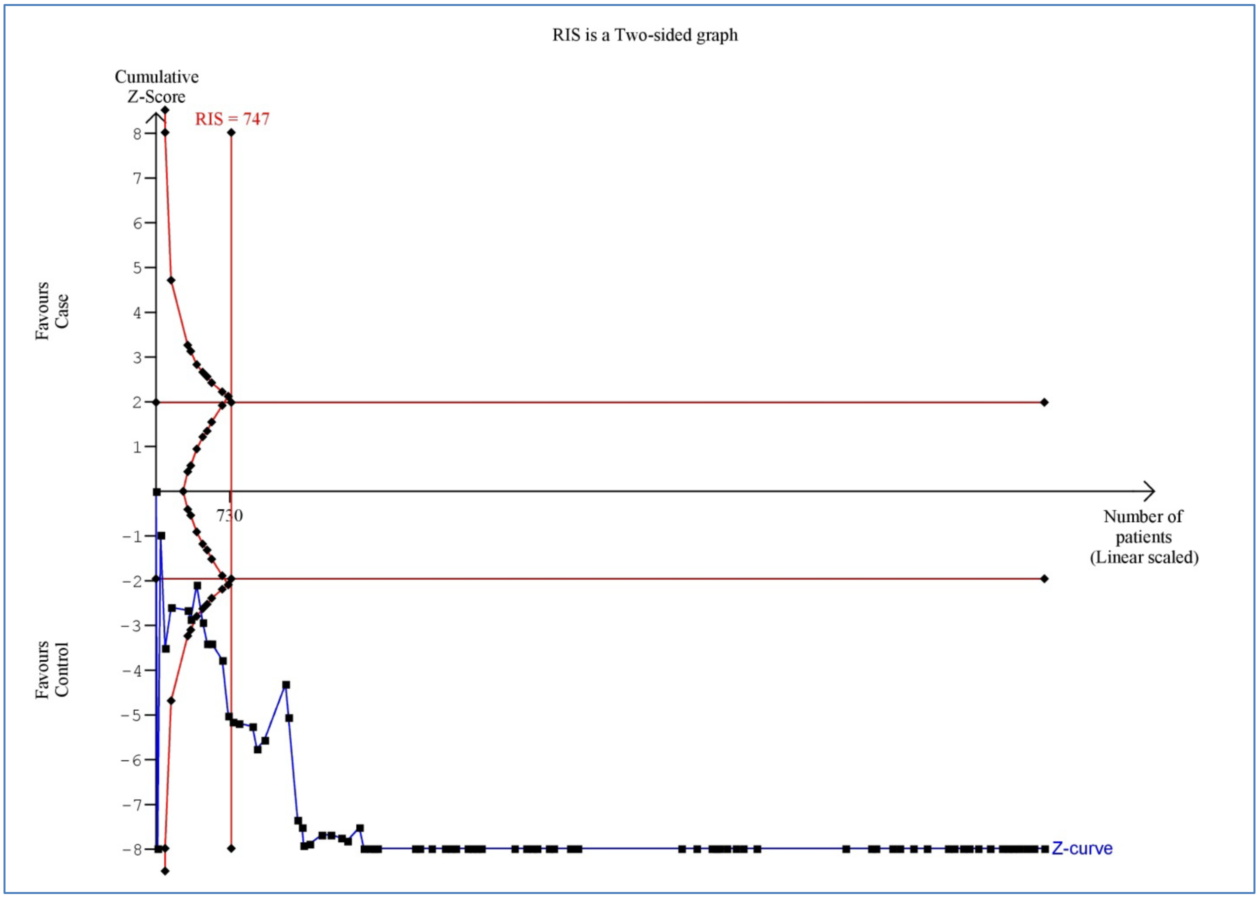

Figure S9: Trial sequential plot analysis of comparison of serum/plasma levels of TNF-α in adults with OSA compared to controls (D^2 = 100%)

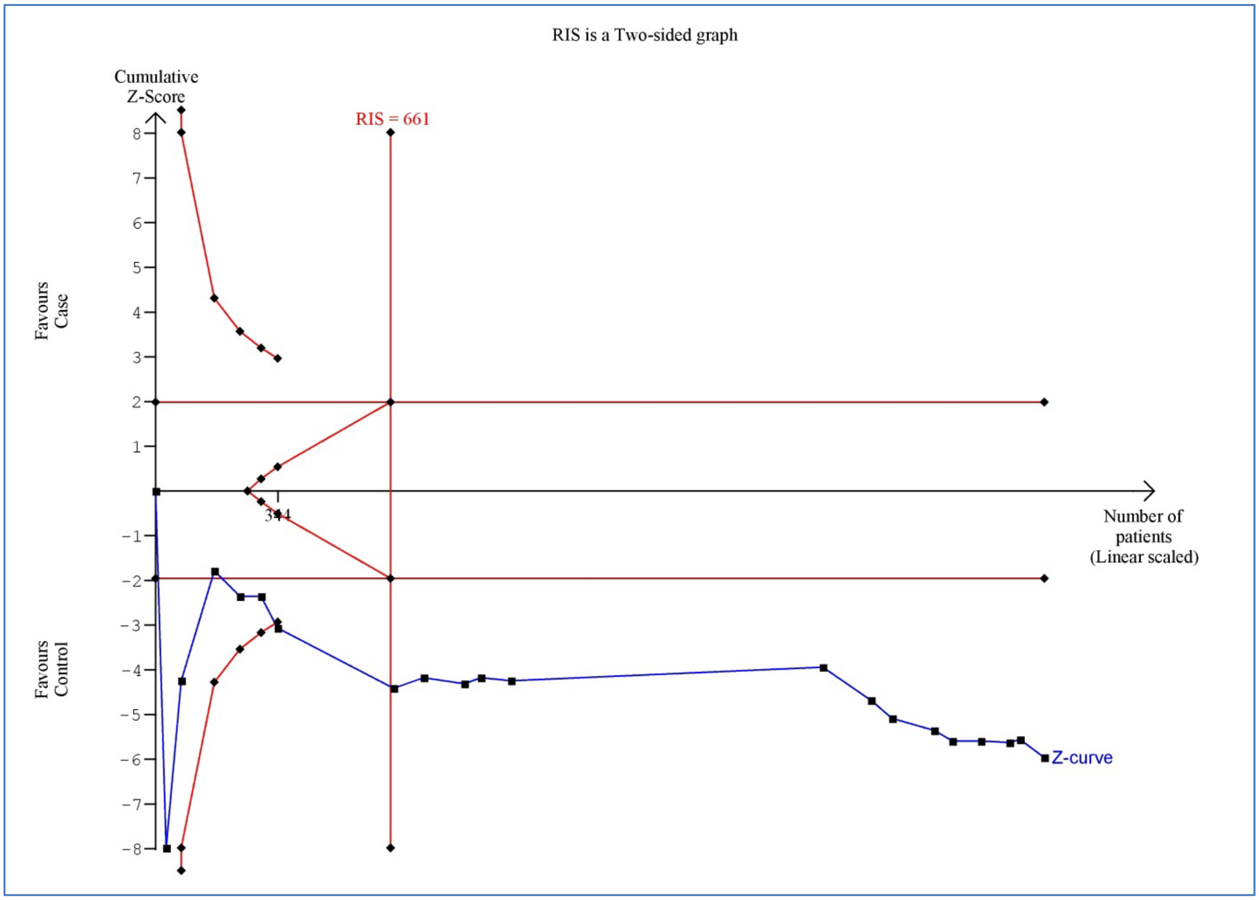

Figure S10: Trial sequential plot analysis of comparison of serum/plasma levels of IL-8 in adults with OSA compared to controls (D^2 = 99%)

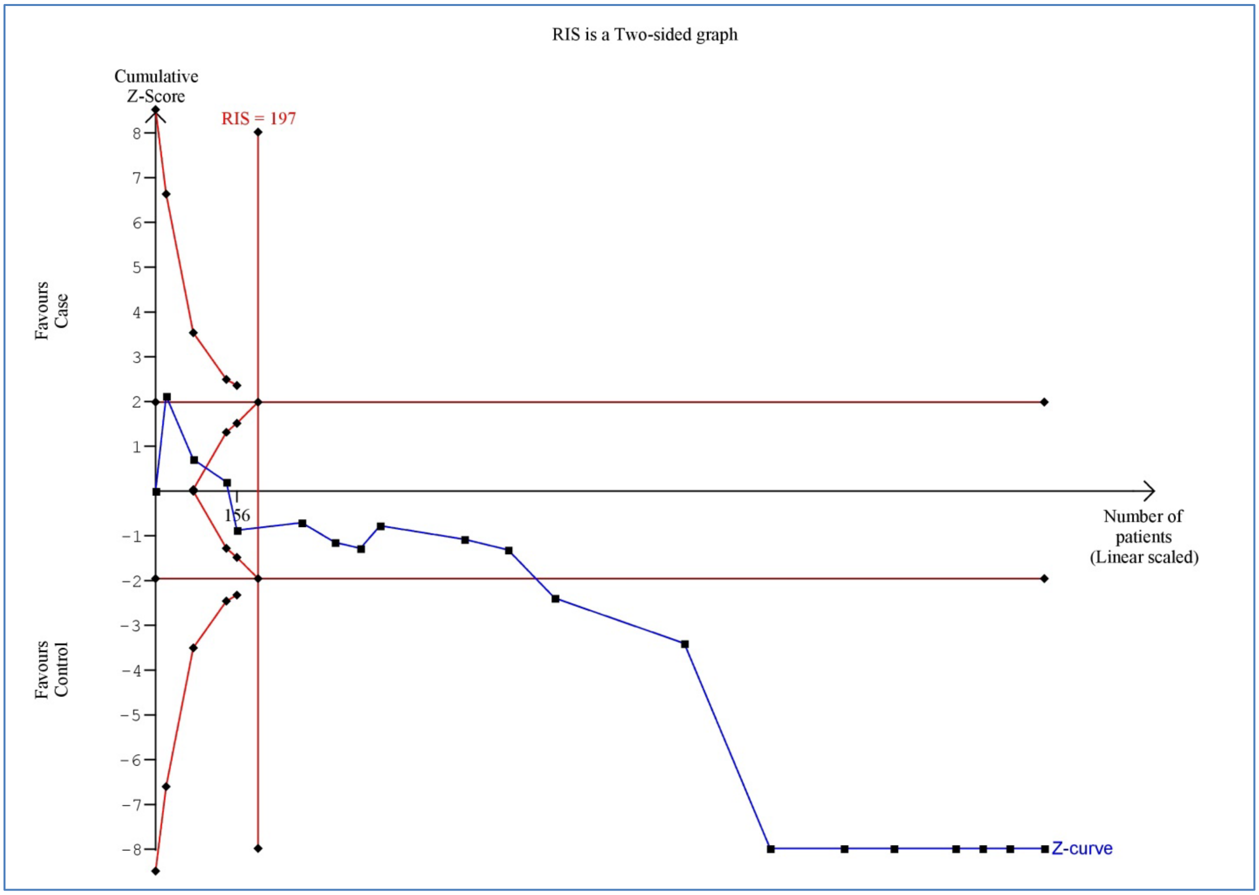

Figure S11: Trial sequential plot analysis of comparison of serum/plasma levels of IL-1β in adults with OSA compared to controls (D^2 = 99%)

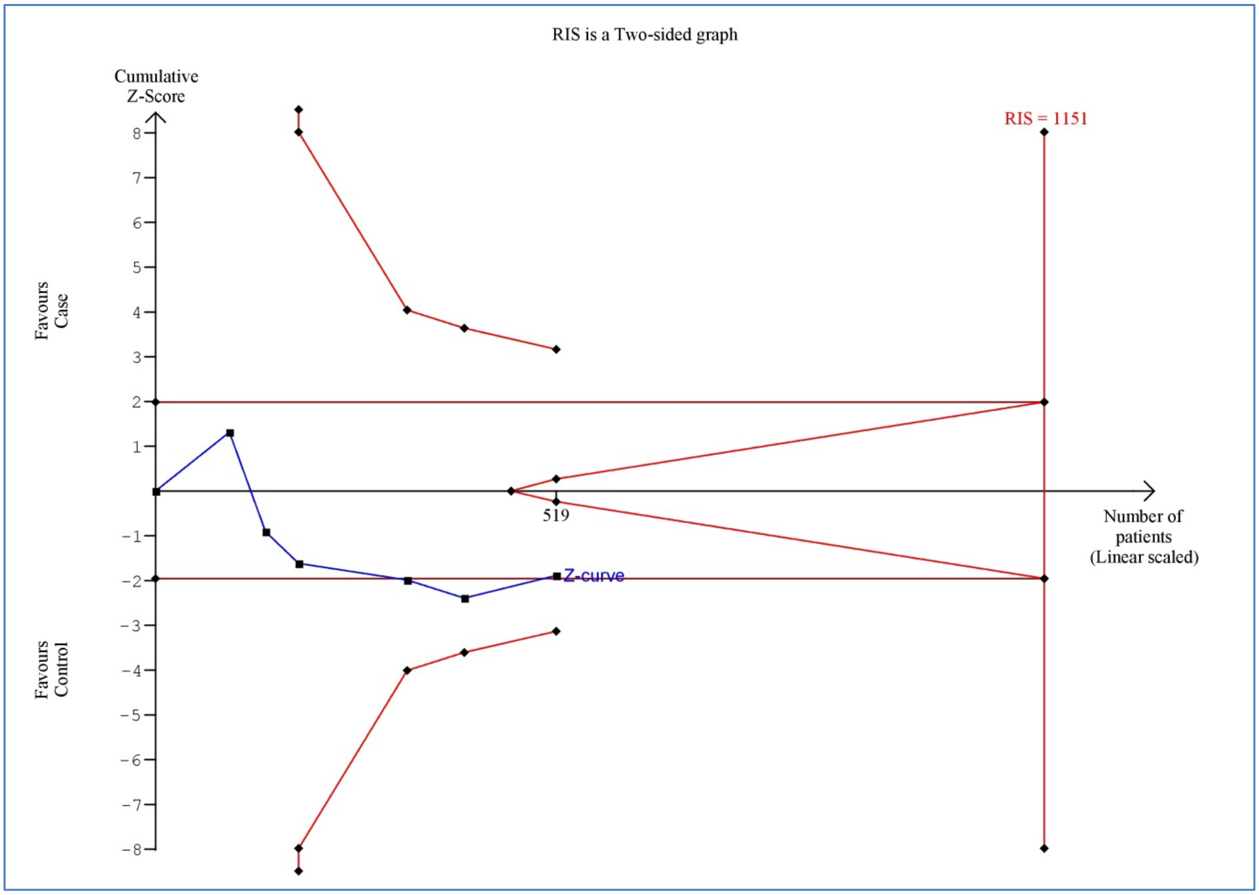

Figure S12: Trial sequential plot analysis of comparison of serum/plasma levels of IFN-γ in adults with OSA compared to controls (D^2 = 97%)

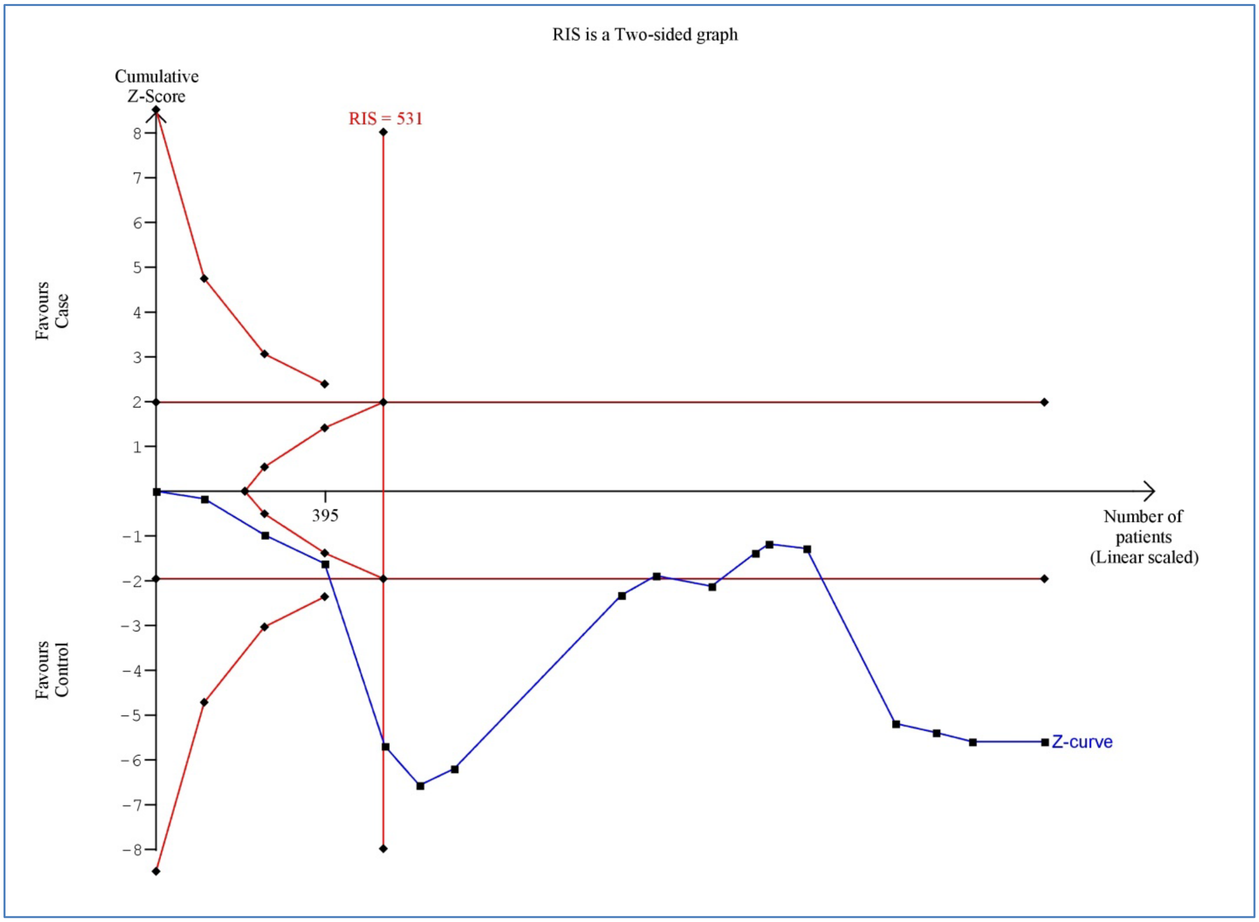

Figure S13: Trial sequential plot analysis of comparison of serum/plasma levels of TNF-α in children with OSA compared to controls ($D^2 = 100\%$)

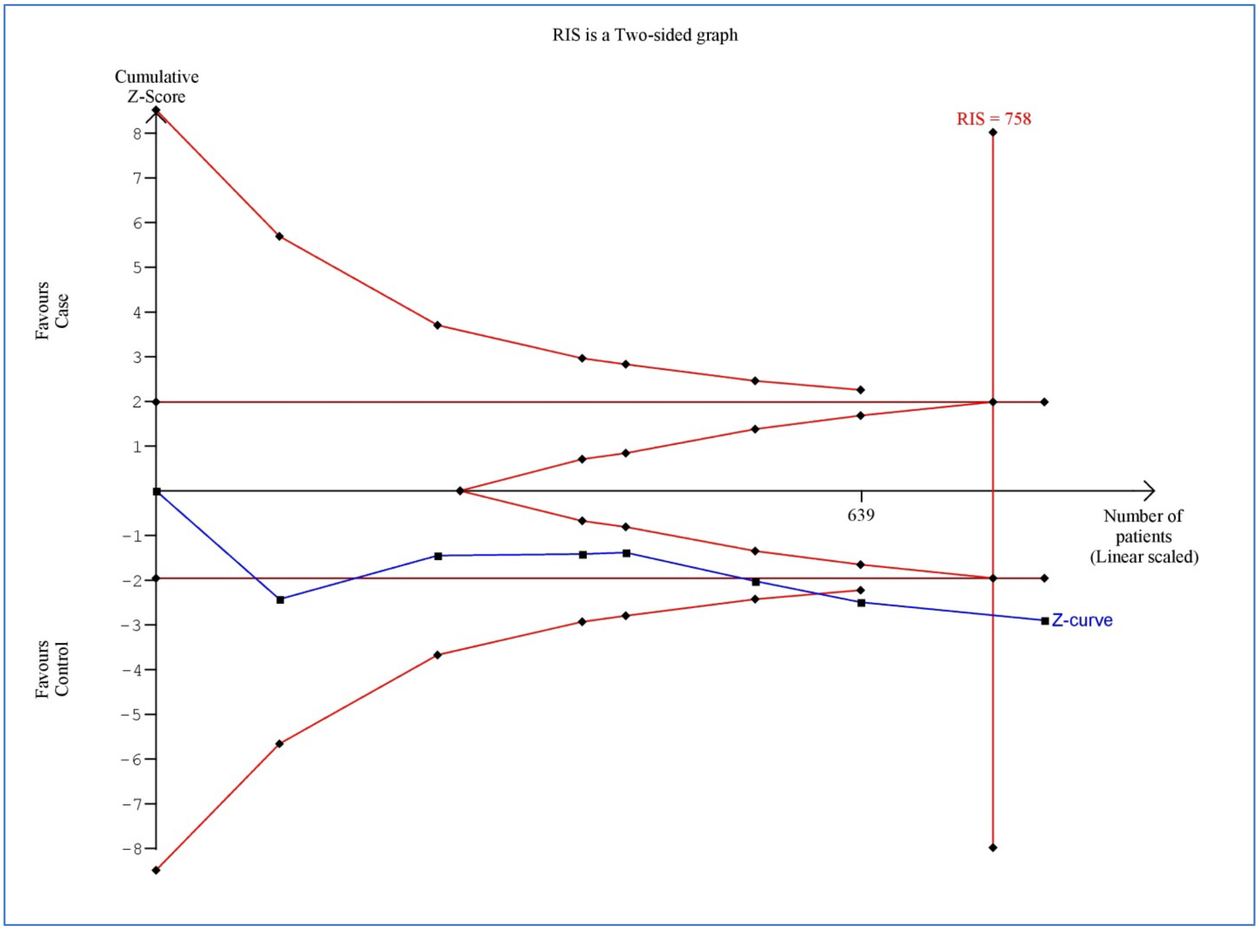

Figure S14: Trial sequential plot analysis of comparison of serum/plasma levels of IL-8 in children with OSA compared to controls (D^2 = 98%)

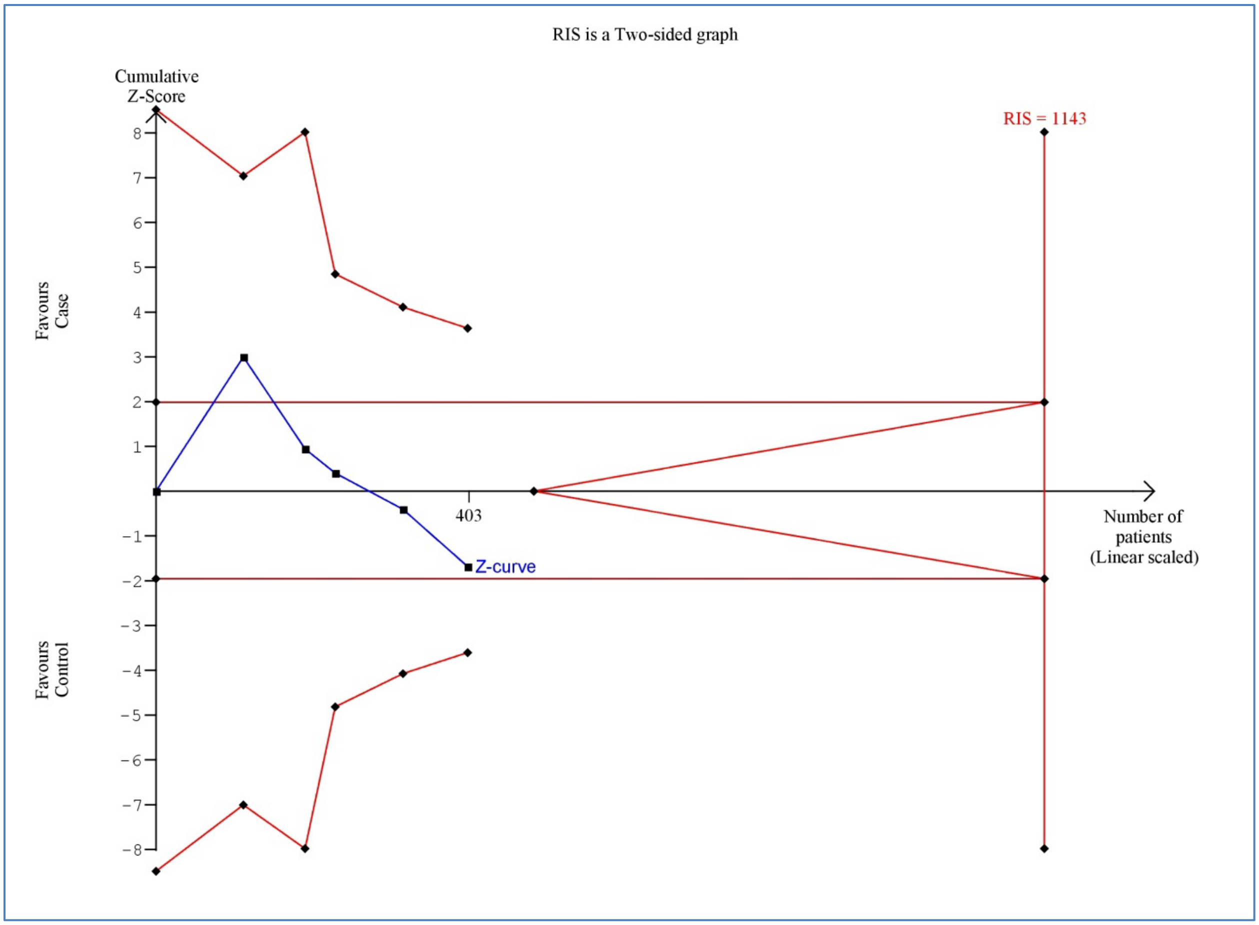

Figure S15: Trial sequential plot analysis of comparison of serum/plasma levels of IL-1β in children with OSA compared to controls (D^2 = 98%)
